# Effective anode materials for *in situ* Sn@C nano-lamellar assembly with doped nanotubes in lithium-ion batteries†

**DOI:** 10.1039/d5ra02378e

**Published:** 2025-06-06

**Authors:** Mengna Xie, Yongheng Zhou, Shuai Dong, Fei Li, Fenghua Zhang, Wei Wei, Jinhai Cui

**Affiliations:** a School of Petrochemical Engineering, Liaoning Petrochemical University Fushun 113001 P. R. China zhangfenghua@lnpu.edu.cn; b Henan Engineering Center of New Energy Battery Materials, School of Chemistry and Chemical Engineering, Shangqiu Normal University Shangqiu 476000 P. R. China cuijinhai535@gmail.com weiweizzuli@163.com; c School of Material and Chemical Engineering, Kaifeng University Kaifeng 475000 P. R. China

## Abstract

Lower lithium-ion diffusion rates and significant volumetric expansion present serious challenges for using SnO_2_/SnO composites as promising anode materials in advanced lithium-ion batteries. To address this issue, we synthesized a novel Sn@C/CNT composite from a Sn-based organometallic complex with 2-methylimidazole and oxidized multi-wall carbon nanotubes. Structural analysis has confirmed that the tin-based composites consist of nano-lamellar assemblies modified by oxidized carbon nanotubes. In these composites, the tin active particles have an average size ranging from 2 to 3 nm, while the layered nano-lamellar structure has an average thickness of 6 nm. The resulting Sn@C/CNT anode material demonstrated a stable specific capacity of up to 688 mA h g^−1^ even after 500 cycles at a higher charging–discharging current density of 1 A g^−1^. The significant diffusion-controlled lithium ion diffusion coefficient of approximately 10^−12^ cm^2^ s^−1^ indicates vigorous dynamic activity from reversible Sn–Li alloy electrochemical reactions. Additionally, the substantial capacity-controlled lithium ion diffusion coefficient, which drops to 10^−16^ cm^2^ s^−1^, illustrates the predominance of the pseudo-capacitance arising from interface reaction. By coupling electrochemical impedance spectroscopy, galvanostatic intermittent titration technique, and linear sweep voltammetry, the mixed lithium-ion diffusion effect was proposed to explain the remarkable adaptability of these Sn-based anode materials for cycling performance across a wide range of specific currents. This work provides a new intention for resolving the drastic volumetric expansion and unsatisfactory dynamic activity of Sn-based anode materials.

## Abstract 2

A unique nano-lamellar structure, combined with diffusion-controlled and capacity-controlled lithium-ion dynamics, achieves remarkable electrochemical performance for this kind of Sn-based anode material in lithium-ion batteries.

## Graphical abstract

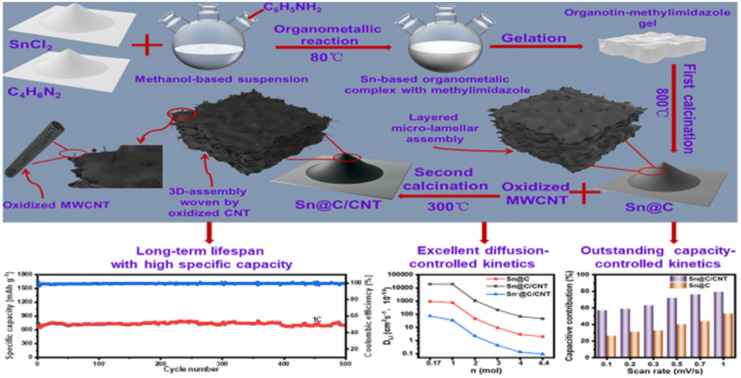

## 1. Introduction

Over the past few decades, significant advancements have been made in the use of lithium-ion batteries (LIBs) as a promising alternative to traditional fossil fuels. These batteries are attractive due to their suitable power density, long cycling life, and zero greenhouse gas emissions.^1–3^ However, the theoretical capacity of commercial graphite, which is currently the primary anode material in LIBs, is only 372 mA h g^−1^, making it insufficient for high-energy storage systems.^4,5^ Therefore, there is ongoing exploration for new cathode materials or innovative combinations of materials that offer higher specific capacities and longer lifespans.^6,7^

Tin-based materials, including Tin (Sn), SnO_*x*_ (where 1 ≤ *x* ≤ 2), and its sulfides, have gained attention as promising anode materials for advanced LIBs due to their high theoretical specific capacity and lower lithiation plateau.^8^ According to research on the interaction between SnO_*x*_ and lithium,^9^ the mechanism by which SnO_*x*_ stores lithium ions has been described as a two-step electrochemical reaction, as outlined by Chen and others.^10^

**Scheme 1:**
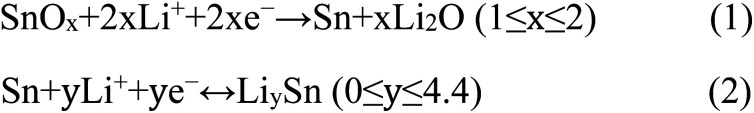
Two-step electrochemical mechanism of Tin oxide with lithium-ion.

Scheme 1 shows those both SnO_2_ and SnO have the maximum theory-specific capacity of either 1494 mA h g^−1^ or 1273 mA h g^−1^, respectively, when alloy Li_*y*_Sn (*y* = 4.4) were obtained in reaction (2), in which electrons *y* can be up to 4.4 (reaction (2)) and has been confirmed by Dahn and Courtney.^11^ Although 783 and 398 mA h g^−1^ of the theory-specific capacity can be pinpointed to either SnO_2_ or SnO, respectively, they seem meaningless because of the irreversible interaction (1) of SnO_*x*_ and Li^+^ in Scheme 1. Therefore, the fundamental theory-specific capacities of those SnO_2_, SnO, and Sn should correspondingly drop down to 783 mA h g^−1^, 876 mA h g^−1^, and 994 mA h g^−1^, respectively, when the alloying reaction reversibly goes on until the final alloy Li_22_Sn_5_ (*y* = 4.4) was obtained (eqn (2)).

However, lower dynamic activity in the diffusion of lithium ions and significant volumetric expansion of the Sn–Li alloy phase during the charging and discharging processes of Sn-based anode materials can lead to a substantially reduced specific capacity. Additionally, this can cause severe efflorescence and abnormal growth of the Solid Electrolyte Interface (SEI), ultimately decreasing the cycling life of LIBs.^12^ So far, exploiting elemental Sn as the anode material in LIBs has been difficult. Researchers have proposed various strategies to address the limitations of Sn-based anode materials.^13^ One effective approach involves minimizing the particle size of these materials. For instance, X. Ye and colleagues reported that they prepared a type of five-nanometer Sn/C composites by annealing Sn-based sludge, which was degraded by microorganisms, under an inert atmosphere. These fine Sn/C nanorod materials exhibit reduced efflorescence, stable cycling life, and excellent rate capability in LIBs.^14^ Another method to enhance the performance of Sn-based anode materials is to create functional composites that combine Sn-based materials with structural carbon sources, such as carbon nanotubes (CNTs), graphene (GN), and three-dimensional (3D) network carbon. Among the various Sn-based composites, Sn/CNT, Sn/GN, and Sn/3D-C composites demonstrate significant resistance to efflorescence and deformation, as well as lower impedance. This is achieved by confining the Sn-based materials within the cavities of these carbon structures,^15,16^ thereby localizing them within their layers^17–19^ and trapping them in lattices.^20^ Consequently, the bulk deformation of these anode composites can be effectively controlled, and their impedance can be reduced.

Unlike the mechanical mixing of carbon nanotubes or graphene sheets, 3D network-carbon composites are typically prepared through *in situ* growth.^21^ This method allows the 3D network-carbon matrix to better embed the Sn-based active materials, resulting in improved conductive capabilities.^22^

In this paper, we present a new network-carbon composites made from Sn-based materials. This innovative approach combines both an *in situ* preparation of 2D nano-lamellar assembly and the mixing preparation of carbon nanotubes, which are annealed at higher temperatures, with carbon nanotube doping, which is annealed at lower temperatures. The goal is to achieve a long-term cycling lifespan of Sn-based anode material by addressing volumetric expansion, enhancing the activity of electro-chemical reactions, and accelerating the migration rate of Li^+^ ions for this Sn-based anode. Additionally, this method offers a novel synthetic routine for other anode materials. To our knowledge, this proposal has not been previously reported.

## 2. Experimental

### 2.1. Preparation of oxidized multi-walled carbon nanotube

Oxidized multiwalled carbon nanotube (MWCNT) (0.2 g, 99%, Shanghai Kelaman Reagent Co., Ltd) was added carefully into a mixed acid solution (40 mL, H_2_SO_4_, 98 wt%; HNO_3_, 65 wt%; *V*_H_2_SO_4__ : *V*_HNO_3__ = 3 : 1) at ambient temperature. The mixture was stirred for 5 hours at 100 °C until it became a dark suspension solution. The brutal acid suspension was fetched out and dropped slowly on the crushed ice to obtain sediment of erosive carbon nanotube. The residue obtained from filtration was dispersed into a glacial acetic acid solution (100 mL, CH_3_COOH, 99 wt%). It was oxidized by a per-acetic acid solution (50 mL, *V*_CH_3_COOH_ : *V*_H_2_O_2__ = 1 : 1) for 2 hours at 80 °C. The residue obtained after filtration and rinse by deionized water was dispersed into an acetone solution of water (C_3_H_6_O, 99.5%; *V*_H_2_O_ : *V*_C_3_H_6_O_ = 1 : 24, 250 mL). The suspension of MWCNT milled by ultrasonic cell Pulverizer(JY92-IIN-YT, Tuohe Electromechanical Technology (Shanghai) Co., Ltd)for half an hour has a molar concentration of 0.67 mmol mL^−1^.

### 2.2 Preparation of tin(ii) complex with 2-methylimidazole

2-Methylimidazole (3 g, C_4_H_6_N_2_, 99 wt%) powder and a tiny amount of phenylamine (1 mL, C_6_H_5_NH_2_, 99 wt%) was dissolved in 150 mL of methanol solution at ambient temperature with stirring. Stannous chloride (3.46 g, SnCl_2_, 99 wt%) was added slowly into this solution, and stirred for 3 hours when reaction temperature has risen to refluxing temperature. The milky suspension finally changed to a transparent methanol solution of quaternary ammonium salt based on the combination of 2-methylimidazole and stannous chloride. The given methanol solution was centrifuged at 1000 rpm for 15 minutes for removing the unreacted stannous chloride. All volatile components in solution containing methanol, HCl and phenethylamine were gradually removed by vacuum rotary evaporation with increased temperature from 30 °C to 45 °C. the milky gel of tin(ii) complex with 2-methylimidazole was collected in a yield of 45 wt%, and the chief component of this complex is SnCl_2_·2(2-methylimidazole) and its dimer, as indicated by the adducts of 1-vinylimidazole, 1-benzylimidazole, and 1,2,4-triazole with tin(ii) chloride reported by vasnin^23^ Except for MWCNT raw, all the above reagents were purchased from Sinopharm Chemical Reagent Com., Ltd

### 2.3 Preparation of *in situ* Sn-based carbon nano-lamellar assembly

The Sn-based 2-methylimidazole compound (1.38 g) was placed in a small porcelain boat wrapped in copper foil and subsequently annealed in a tube furnace at 800 °C for 4 hours. The resulting greyish-black Sn–C composites was collected, yielding 24 wt%. Three Sn–C nano-lamellar assemblies were annealed at temperatures of 700 °C, 800 °C, and 900 °C, and are referred to in this paper as Sn@C^a^, Sn@C, and Sn@C^b^, respectively.

### 2.4 Preparation of *in situ* Sn-based carbon nano-lamellar assemblies doped with MWCNT

The given *in situ* Sn-based carbon assemblies (0.2 g) was added into 25 mL of acetone–water suspension of oxidized MWCNT (0.067 mmol mL^−1^), and milled for 6 hours in a planetary ball mill. The fine sediments of the acetone–water solution were centrifuged at 1000 rpm for 15 minutes, and dried in a vacuum chamber for half an hour. The mixture of *in situ* Sn-based carbon assemblies and the oxidized MWCNT particles was annealed in a tube furnace at 300 °C for 4 hours. The obtained *in situ* Sn-based carbon nano-lamellar assemblies doped by oxidized MWCNT are marked as Sn@C/CNT^a^ Sn@C/CNT, and Sn@C/CNT^b^ known by different annealing temperatures at 700 °C, 800 °C, and 900 °C, respectively.

With the comparison, the commercial acetylene black (35–45 nm) and nano Tin (100 nm in size, Shanghai Pantian Powder Materials Co., Ltd) were repeatedly mixed in a same dosage of the acetone–water solution, milled, annealed at 800 °C for getting the *in situ* Sn-based carbon nano-lamellar assemblies, and then doped with the same amount of oxidized MWCNT, dealt with as same as those processes of Sn@C/CNT for getting the *in situ* Sn-based carbon assemblies doped by MWCNT. The obtained composite is marked as Sn*@C/CNT.

### 2.5 Material characterizations

The crystalline structure of the composites was analyzed by Philips X'pert X-ray diffractometer with Cu Kα radiation at a scanning rate of 5^°^ min^−1^ in the range of 10–80° (XRD). The morphology and microstructure of composites were characterized by Thermo Fisher Verios G4 Scanning electron microscopy (SEM), Thermo Fisher Verios G4 energy dispersive X-ray spectroscopy (EDS), and JEM-2100 JEOL transmission electron microscopy (TEM), respectively. Shimadzu DTG-60/DTG-60A Thermogravimetric and differential thermal analysis (TG-DTA) finished the carbon content and structure characteristic of prepared Sn-based composites. Thermo VG ESCALAB 250 X-ray photoelectron spectroscopy (XPS) was used to investigate the chemical composition and elemental valence states of three tin-based materials based on different binding energy (BE) peaks. Nitrogen adsorption–desorption isotherms, Barret–Joyner–Halenda (BET) specific surface area, Barret–Joyner–Halenda (BJH) pore size distribution, and Horvash–Kawazoe (HK) micro-pore analysis were performed by 3H-2000PM high performance specific surface and porosity analyzer. Raman spectra were collected to analyzed the functional groups of three Sn-based composites on a Invia Qontor spectrometer.

### 2.6 Electrochemical measurement

The assembled CR 2032 coin-type batteries underwent a series of electrochemical performance tests. The active materials, consisting of acetylene black and polyvinylidene fluoride (PVDF), were hand-ground using a pestle in a jade mortar at a weight ratio of 8 : 1 : 1 for a duration of 0.5 hours. A few drops of *N*-methyl-2-pyrrolidone (NMP) were added to create a uniform slurry, which was then coated onto a copper foil base and dried overnight in a vacuum oven at 120 °C. The coated copper foil was cut into discs with a diameter of 14 mm to serve as working electrodes. The typical active material load ranged from 1 to 1.5 mg cm^−2^ (including the weight of acetylene black and the adhesive). Next, a pure lithium sheet was used as the negative electrode, a Celgard 2400 polypropylene porous membrane served as the diaphragm, and lithium-ion electrolyte (LB-141) was used as the electrolyte. The half battery was then assembled in a Vigor glove box filled with high-purity argon, ensuring that the oxygen and water levels were below 2 ppm. LAND CT2001A battery Test system performed charging–discharging measurements of lithium ion at room temperature in a fixed voltage range of 0.01 to 3.0 V. CHI660E electrochemical workstation with a three-electrode system was used to perform cyclic voltammetry (CV), electrochemical impedance spectroscopy (EIS), galvanostatic intermittent titration technique (GITT), and linear sweep cyclic voltammetry (LCV) tests.

## 3. Results and discussion

### 3.1 Structural analysis for anode materials

**Fig. 1:**
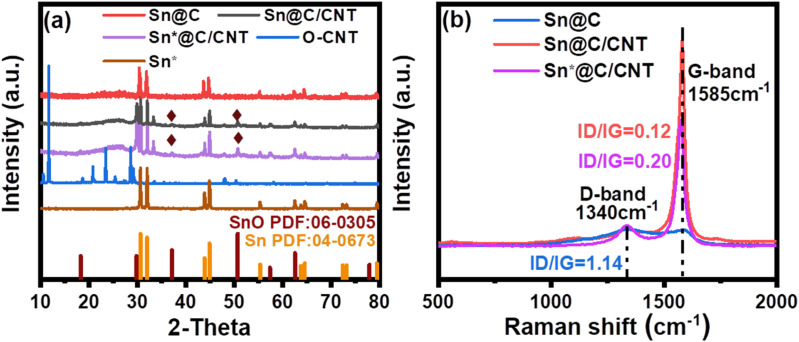
XRD patterns (a) for Sn@C, Sn@C/CNT, Sn*@C/CNT, oxidized carbon nanotube (O-CNT), and nano Tin (Sn); and Raman spectra (b) for Sn@C, Sn@C/CNT and Sn*@C/CNT materials.

XRD patterns and Raman spectra of Sn@C, Sn@C/CNT, and Sn*@C/CNT are shown in Fig. 1(a) and (b).

Fig. 1(a) presents the XRD diffraction peaks of the Sn@C nano-lamellar assemblies, which display significant intensity at 2*θ* values of 30.02, 30.64, 44.90, and 55.33^°^. These characteristic peaks, along with several smaller peaks, align with the distinctive lines of the ICDD standard card PDF#04-00673 for tetragonal crystal Sn. This indicates that Sn^2+^ has been completely reduced to Sn after the *in situ* Sn-based carbon assemblies was annealed at 800 °C in a nitrogen atmosphere. The lattice parameters for the resulting white tin correspond to *a* = *b* = 5.83 Å and *c* = 3.18 Å. Whatever, the *in situ* Sn-based carbon annealing at either 700 °C (Sn@C^a^ and Sn@C/CNT^a^) or 900 °C (Sn@C^b^ and Sn@C/CNT^b^) exhibit either the increased Sn content or the formation of the Sn phase with larger particle sizes in the complexes (see Fig. S1(a) and (b)†). These will ultimately impair their cycling performance in LIBs. The XRD pattern of the Sn@C/CNT composites also indicates that the addition of MWCNT does not alter the crystal structure of Sn, but there is a minor presence of SnO as an impurity. This impurity is confirmed by two diffraction peaks at 2*θ* values of 37.15 and 50.76°, which correspond to the characteristic lines noted in the ICDD standard card PDF#06-0305 for SnO. The presence of hydroxyl or carboxyl groups in the oxidized CNT can further oxidize Sn. The same SnO impurity is also observed in the XRD patterns of both Sn@C/CNT^a^ and Sn@C/CNT^b^ (see Fig. S1† (b)). In comparison with the XRD patterns of the purely commercial Tin particles, Sn*@C/CNT obtained through the addition of acetylene black (annealed at 800 °C) and CNT (annealed at 300 °C) to commercial tin nanoparticles sized 100 nm also displays the presence of SnO impurity. This observation indicates that the impurity arises from the oxidation process between the commercial Tin particles and the oxidized MWCNT. Furthermore, the oxidized CNT material displays acute peaks at 2*θ* values of 11.65, 23.48, and 28.60, alongside a large, broad XRD diffraction peak at 2*θ* = 26.62°. This broad peak indicates that both the oxidized CNT and the *in situ* carbon composites possess a crystal structure similar to that of graphite.^24^ The ordered carbon structures present in both the tin-situ graphite and oxidized CNT are expected to enhance the electrochemical performance of the Sn-based anode materials.

Fig. 1(b) illustrates the relationship between the D-band peak and the G-band peak in the Raman spectra for three Sn-based carbon materials. The D-band peaks, associated with the stretching vibrations of C–C and C=C bonds, are observed at 1340 cm^−1^. The intensity of these peaks correlates with lattice defects, structural distortions, and the amorphous state of carbon atoms.^25^ In contrast, the G-band peaks, which result from the in-plane stretching vibrations of sp^2^ hybrid carbon atoms, are located at 1585 cm^−1^. These peaks reflect the stratification and topological structures of carbon, such as graphene and graphite.^25^ The *I*_D_/*I*_G_ value of 1.14 indicates that the Sn@C assembly contains a significant amount of *in situ* carbon materials, which exhibit a higher number of lattice defects, structural distortions, and an amorphous state of carbon atoms. This condition enhances the internal activity of both carbon matrix and Sn nano particles to combine the lithium ions.^26^ Reducing the *I*_D_/*I*_G_ ratio by enhancing the structure regularity of the Sn@C nano-lamellar assembly is essential to balance the electronic conductivity and the diffusion efficiency of lithium ions. Lower values of *I*_D_/*I*_G_ realized by incorporating the oxidized MWCNT, precisely 0.12 for Sn@C/CNT and 0.20 for Sn*@C/CNT shown in Fig. 1(a), are expected to achieve this purpose.

**Fig. 2:**
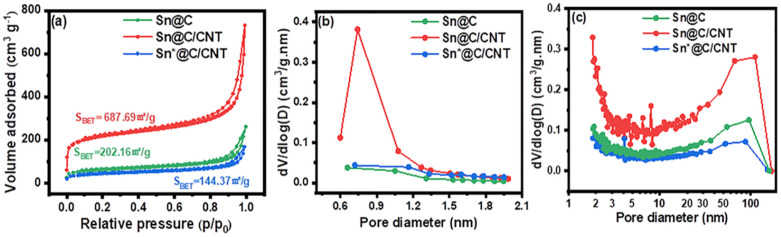
(a) N_2_ adsorption–desorption isotherms, (b) HK micro-pore analysis and (c) pore size distributions and specific surface area of Sn@C, Sn@C/CNT and Sn*@C/CNT.

The BET adsorption–desorption isotherms, HK micropore analysis, and BJH pore size distributions for Sn@C, Sn@C/CNT, and Sn*@C/CNT are presented in Fig. 2. The pronounced hysteresis loop observed in each adsorption–desorption isotherms shown in Fig. 2(a) indicates that all three materials possess a mesoporous structure.^27^ In Fig. 2(b), it is evident that Sn@C/CNT exhibits a significantly higher value of d*V*/d log *D* = 0.382 cm^3^ g^−1^ nm^−1^ in the HK micropore analysis, while Sn@C and Sn*@C/CNT only show much lower values of 0.038 cm^3^ g^−1^ nm^−1^ and 0.043 cm^3^ g^−1^ nm^−1^, respectively. This suggests that the Sn@C/CNT composites contains nearly ten times more micropores compared to the other two composites.

The BJH pore size distributions of three composites at different scales are illustrated in Fig. 2(c) and summarized in Table 1. The results indicate that the micro-pores and meso-pores in the Sn@C/CNT composites contributed the most significantly to the total specific surface area, which measured 687.69 m^2^ g^−1^, accounting for 72.8%. In comparison, the Sn@C nano-lamellar assembly had a total specific surface area of 202.16 m^2^ g^−1^, contributing 62.4%, while the Sn*@C/CNT sample had 144.37 m^2^ g^−1^, contributing 62.3%. Additionally, the Sn@C/CNT composite features the smallest average pore size of 6.09 nm and the largest *D*_90_ hole diameter of 58.69 nm among the three composites, further supporting these observations. Overall, the combination of the largest surface area (687.69 m^2^ g^−1^), significant micro-pore contributions, and the smallest average pore diameter in the Sn@C/CNT composites may play a crucial role in effectively coating the nano Sn particles, accommodating the bulk expansion of Sn, and facilitating the diffusion rate of Li^+^.^28^

**Table 1:** BJH Pore size distributions and BET specific surface area of three anode materials

Samples	SA^a^ (m^2^ g^−1^)	MSA^b^ (m^2^ g^−1^)	APD^c^ (nm)	*D*_90_ d (nm)	Pore volume (ml g^−1^)
Sn@C	202.16	126.89	7.19	37.59	0.36
Sn@C/CNT	687.69	500.73	6.09	58.69	1.05
Sn*@C/CNT	144.37	90.49	7.18	51.67	0.26

a Specific area.

b Micro-pore specific area.

c Average pore diameter.

d 90^th^ percentile diameter.

**Fig. 3:**
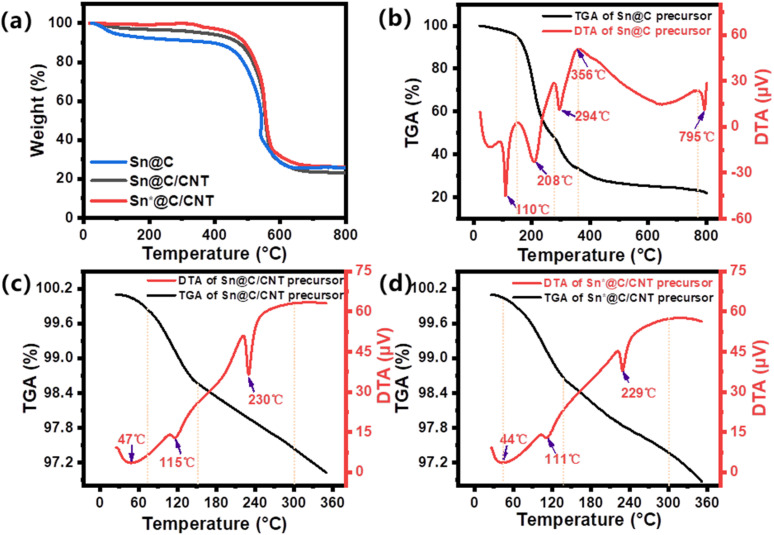
(a) TG-DTA curves of the three anode materials of Sn@C, Sn@C/CNT, and Sn*@C/CNT under air; TG-DTA curves of (b) Sn@C precursor, (c) Sn@C/CNT precursor, and (d) Sn*@C/CNT precursor in N_2_ atmosphere.

TGA and DTA curves of three composites are presented in Fig. 3(a–d). The Sn content for the three composites shown in Fig. 3(a) is 20.3 wt%, 18.3 wt%, and 26.2 wt%, respectively. The post-weight of the materials annealed in a furnace tube under an air atmosphere should be converted from that of SnO_2_ to the weight of pure Sn. In Fig. 3(b), the Sn@C precursor displays three exothermic peaks at 110, 208, and 294 °C. The first exothermic peak at 110 °C corresponds to the release of heat from water, while the subsequent peaks at 208 °C and 294 °C indicate heat loss associated with the breakdown of 2-methylimidazole ligands.^29^ Notably, the additional endothermic peak observed at 356 °C not only marks the onset of the crystallization of Sn but also signifies the reduction of Sn^2+^ due to the reductive gases released during ligand degradation. In Fig. 3(c) and (d), further annealing of the Sn@C and Sn*@C at 360 °C in a nitrogen atmosphere was conducted with the addition of oxidized MWCNTs. The DTA of both the Sn@C/CNT and the Sn*@C/CNT precursors show weight loss temperatures around 110 °C and 229 °C, respectively. These temperatures correspond to the release of water molecules from the surface of the oxidized MWCNTs and the decomposition of oxygen-containing functional groups such as carboxylic acids and hydroxyls present in the oxidized MWCNTs.^30^ This indicates that the Sn-based carbon nano-lamellar assembly interacts with the oxidized MWCNTs through decomposition to form a new three-dimensional Sn-carbon materials.

**Fig. 4:**
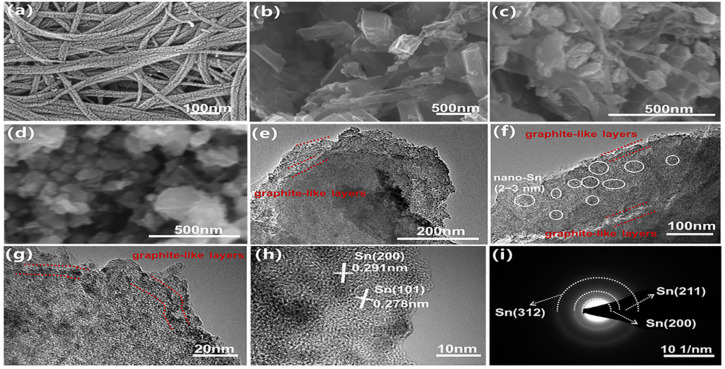
SEM and TEM images of oxidized carbon nanotube and three Sn-based composites. (a) TEM of oxidized carbon nanotube, (b) SEM of Sn@C, (c) SEM of Sn@C/CNT, (d) SEM of Sn*@C/CNT, (e and f) TEM of graphite-like layer Sn@C/CNT, (g and h) HRTEM of Sn@C/CNT, (i) corresponding micro-area diffraction pattern of Sn@C/CNT in HRTEM.

Fig. 4 illustrates the topographies of oxidized carbon nanotube, Sn@C, Sn@C/CNT, and Sn*@C/CNT materials. Fig. 4(a) highlights the distinct cracks that appear on the wall of the carbon nanotube following the oxidation process. These oxidized cracks can provide more edge carbon atoms, increasing the amount of the naked functional groups (*e.g.*, COOH, OH), and enhancing the compatibility between carbon nanotubes and Sn@C material.^31^ In contrast to the spherical nano Sn embedded in a disordered carbon matrix found in the Sn*@C/CNT particles (Fig. 4(d)), the Sn@C particles resemble graphite-like nano-lamellar assemblies, as shown in Fig. 4(b). This thin graphite-like slice has an average thickness of only 6 nm (see Fig. S3†). Furthermore, Fig. 4(c) illustrates that these lamellar assemblies of Sn@C particles are bound by doped, oxidized MWCNT to form the new Sn@C/CNT particles. This design aligns with our experimental goal of enhancing the conductivity of the Sn@C composites. EDS observations and corresponding mappings of the Sn@C particles reveals the presence of Nitrogen (N) in the Sn@C material, which can be reasonably attributed to the incomplete oxidation of amino groups in the presence of Sn^2+^ (see Fig. S4†). The EDS analysis and mappings of Sn@C/CNT particles (see Fig. S5†), indicate the presence of oxygen (O) alongside the Tin (Sn) and nitrogen (N); this O contributes to the tiny SnO impurities observed in the composites. Both the Sn and N elements in the Sn@C particle, as well as the Sn, N, and O elements in the Sn@C/CNT particle, are uniformly distributed throughout their carbon-based composites. In contrast, the elements in the Sn*@C/CNT particle are unevenly distributed (see Fig. S6†). This suggests that the simple mechanical mixing of commercial nano-Sn with both acetylene black and oxidized MWCNT cannot ensure structural consistency in the Sn*@C/CNT composites. As a result, we can predict poor electrochemical performance for lithium-ion storage and diffusion. Additionally, the Sn content in both the Sn@C and Sn@C/CNT closely matches the calculations obtained from the TGA results (see Fig. 3(a)).

Fig. 4(e and f) displays the TEM images of the Sn@C/CNT particles. In these images, the bright area represents the carbon matrix, while the small dark spots correspond to the Sn nanoparticles. It is evident that the Sn nanoparticles, which average between 2 and 3 nm in width, are well dispersed within the carbon matrix. Additionally, the lamellar structure of the *in situ* carbon matrix can also be observed together with the that of HRTEM image of Fig. 4(g). Fig. 4(h) presents a HRTEM image of the Sn nanoparticles within the Sn@C/CNT composites. This image reveals lattice fringe spacings of 0.278 nm and 0.291 nm, which correspond to the (101) and (200) planes of the tetragonal Sn phase, respectively. Furthermore, the micro-area diffraction pattern diffraction patterns of the Sn phase, shown in the Fourier Transform HRTEM image (Fig. 4(i)), display perfect concentric-circle patterns. This observation indicates that various clusters of the tetragonal crystalline Sn phase are arranged in an unordered manner, resulting in a polycrystalline system. Within this system, additional crystal planes, such as (211) at the secondary ring and (312) at the outer ring, are clearly visible.

**Fig. 5:**
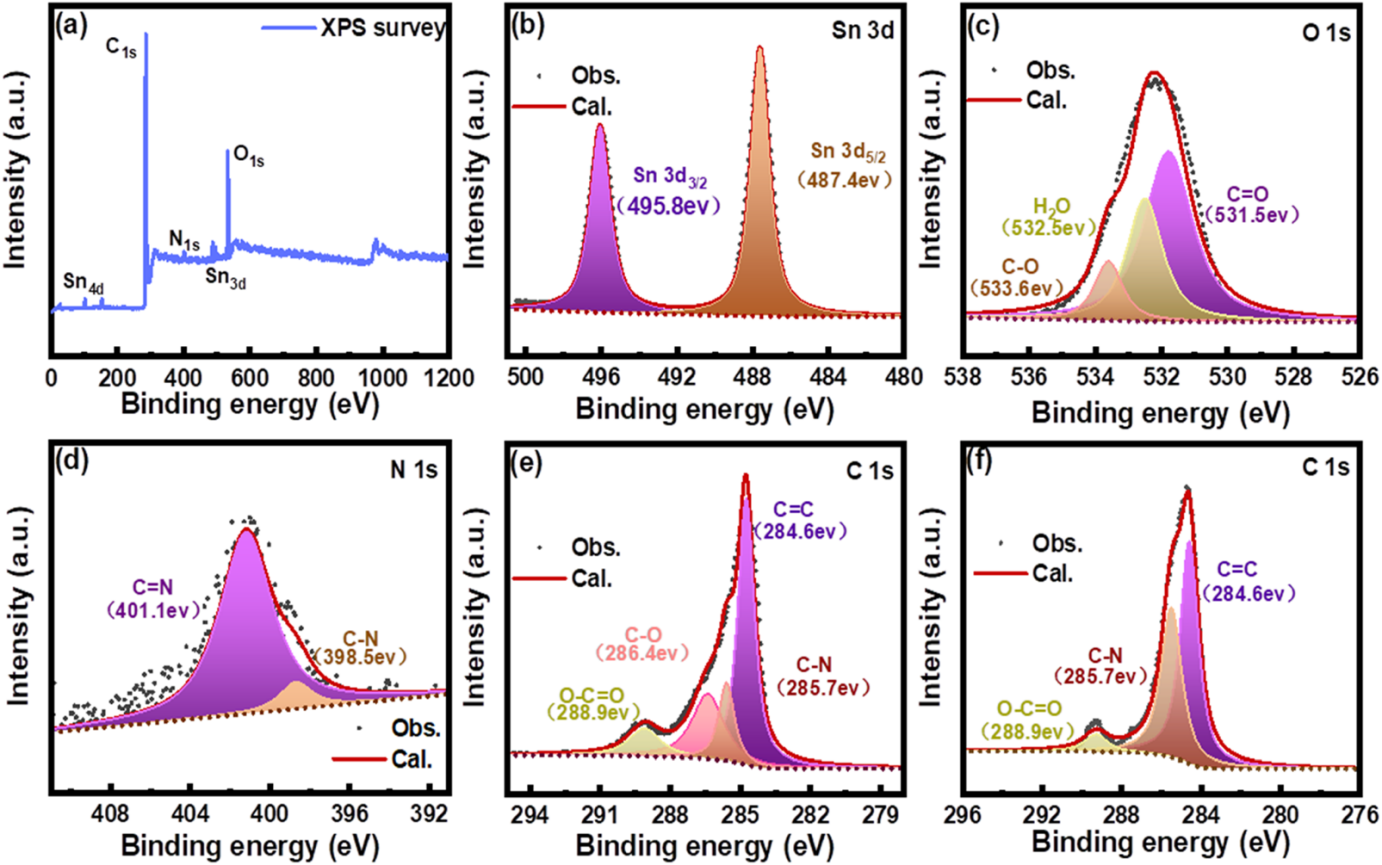
XPS spectra of Sn@C/CNT: (a) survey and deconvoluted, (b) Sn 3d, (c) O 1s, (d) N 1s, (e) C 1s; and of (f) Sn@C C 1s.

The XPS survey spectra of the Sn@C/CNT composites shown in Fig. 5 confirms the presence of tin (Sn), carbon (C), oxygen (O), and nitrogen (N). The Sn 3d spectra (Fig. 5(b)) show only two BE peaks at 487.7 eV and 495.8 eV. These peaks correspond to Sn 3d_5/2_ and Sn 3d_3/2_ of tin, as reported in the literature.^32^ In the O 1s spectrum of Sn@C/CNT (Fig. 5(c)), the observed BE peak splits into three simulated peaks at 531.5 eV, 532.5 eV, and 533.6 eV, which can be assigned to different categories of oxygen: carboxyl (–C=O), hydroxyl (H_2_O), and ether groups (C–O–), respectively.^33^ This also indicates that the oxidized MWCNT can connect to the *in situ* carbon assemblies through hydroxyl groups. The two BE peaks of the N 1s spectrum for Sn@C/CNT, located at 398.5 eV and 401.1 eV, as shown in Fig. 5(d), indicate the presence of both imino and amino groups within the *in situ* carbon assemblies.^34^ This formation occurs when methylimidazole is reduced by Sn^2+^ during the annealing process. The C 1s spectrum of Sn@C/CNT, shown in Fig. 5(e), displays four BE peaks at 284.6, 285.7, 286.4, and 288.9 eV, as determined through deconvolution analysis. These peaks correspond to various carbon categories, including the graphite structure (sp^2^ C=C), nitrile groups (C–N), ether groups (C–O), and carboyl groups (C=O).^35^ Unlike the XPS spectra of Sn@C/CNT, the Sn@C material only exhibited peaks for Sn, N, and C (see Fig. S7†). The C 1s and N 1s spectra for Sn@C showed BE peaks consistent with those observed in Sn@C/CNT. Two deconvoluted BE peaks, depicted in Fig. 5(f), are located at 284.6 eV, corresponding to the signature of graphite (sp^2^ C=C) and amino groups, respectively. Additionally, the BE peak at 288.9 eV can be attributed to the unavoidable absorption of oxygen by the active carbon matrix of Sn@C material.^36^ The XPS spectra of Sn*@C/CNT (see Fig. S8†) did not confirm the presence of nitrogen (N) in the composites, except for the presence of tin (Sn), carbon (C), and oxygen (O). This finding further proved that the source of carbon defects only originates from the annealing process of tin(ii) complex with 2-methylimidazole rather than oxidized MWCNT, which has significant implications for our understanding of the material's properties.

### 3.2 Long-term cycling electrochemical performances

**Fig. 6:**
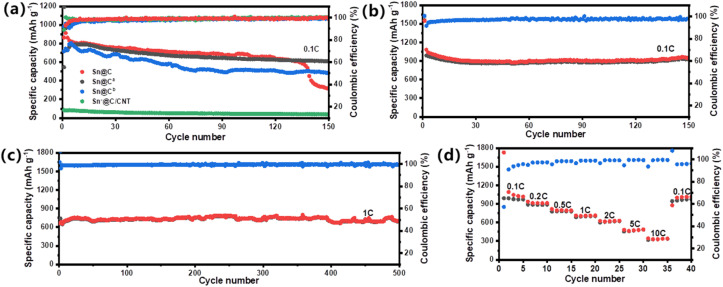
Electro-chemical lithium storage performance: (a) efficiency and long-term cycling performance for Sn@C, Sn@C^a^ (annealed at 700 °C), Sn@C^b^ (annealed at 900 °C), and Sn*@C/CNT at a current density of 100 mA g^−1^; (b) and (c) efficiency and long-term cycling performance for Sn@C/CNT anode material at a current density of 100 and 1000 mA g^−1^, respectively; (d) rate capabilities of Sn@C/CNT anode material from current density of 0.1 to 10 A g^−1^. * All the testing were finished over 0.01–3.0 V at 20 °C.

Fig. 6(a) shows that three *in situ* Sn-carbon materials (Sn@C, Sn@C^a^, and Sn@C^b^) and a doping Sn-carbon material (Sn*@C/CNT) maintain 516.0, 638.4, 688.2, and 44.5 mA h g^−1^ discharging specific capacities, respectively, and almost 99.2% of coulombic efficiency even after 100 cycles. In comparison to the lowest retained specific capacity of the Sn*@C/CNT anode material, the Sn@C material maintains at least 85.6% of its theoretical specific capacity (994 mA h g^−1^) after 100 cycles at a rate of 0.1C. However, the reversible capacity retention rate for the Sn@C anode materials significantly drops to 42% after 150 cycles while anode materials of Sn@C^a^ and Sn@C^b^ drop to 75% and 60%, respectively. This vast decay in retained capacity might contribute to the higher value of *I*_D_/*I*_G_ as was discussed in Raman spectra analysis, in which the excessive carbon defects might ruin the dynamic balance between reaction and diffusion to lithium ion. To enhance the long-term cycling performance of the Sn@C anode material, an improved synthesis strategy was developed for the Sn@C/CNT composite, which now serves as a new anode material. As shown in Fig. 6(b), the Sn@C/CNT maintains a high specific capacity of up to 946 mA h g^−1^, with a capacity retention ratio of 95.6% and a coulombic efficiency of nearly 99.5%, even after 150 charge/recharge cycles at a current density of 100 mA g^−1^. Moreover, after approximately 500 charge/recharge cycles at a higher current density of 1 A g^−1^ (1C), Fig. 6(c) shows that the Sn@C/CNT still retains a specific capacity of 688 mA h g^−1^, demonstrating a capacity retention ratio of 95.0% and a coulombic efficiency close to 99.5%. Fig. 6(d) illustrates the rate capabilities of the prepared Sn@C/CNT across various current densities, ranging from 0.1 to 10 A g^−1^. The Sn@C/CNT anode material achieves average reversible discharge capacities of 997.3, 911.4, 794.4, 708.8, 614.7, 476.8, and 341.4 mA h g^−1^ at current densities of 0.1, 0.2, 0.5, 1, 2, 5, and 10 A g^−1^, respectively. When the current density is returned to 0.1 A g^−1^, a stable discharge capacity of 969 mA h g^−1^ can be restored. This indicates that the Sn@C/CNT exhibits excellent capacity recovery performance even after enduring high current density of up to 10 A g^−1^. For comparison, two other anode materials, Sn@C/CNT^a^ and Sn@C/CNT^b^, first annealed at 700 °C and 900 °C, respectively, show inferior long-term cycling performance and rate capabilities, as illustrated in Fig. S9.† This further demonstrates that the annealing temperature significantly impacts their electrochemical performance.

The impressive cycling performance and rate capabilities can be attributed to the effective activation of the Sn–Li alloy reaction and the excellent pathways for lithium ion diffusion. The three-dimensional carbon network, formed from a layered *in situ* nano-carbon matrix woven with oxidized MWCNTs, can effectively accommodate the significant volume changes of Sn particles. This structure promotes the activation of the Sn–Li alloy reaction and maintains the structural integrity of the anode materials during the lithiation and delithiation processes.

The following summarizes the electrochemical performance of various valuable anode materials, including metallic Sn and functional matrix materials, along with Sn@C/CNT, as presented in Table 2.

**Table 2:** Electrochemical performance for different Sn-base anode materials in LIBs

Materials	Cycling stability/cycles/current density (mA h g^−1^/cyc^e^/A g^−1^)	Rate capability/current density (mA h g^−1^/A g^−1^)	Year	Ref.
Sn-CNTs	437/100/0.1	429/2	2013	21
Sn/MoS_2_/C	625/500/1	630/2	2015	37
Mn_2_SnO_4_/Sn/C	908/100/0.5	550/2	2016	38
Sn-PMA^a^	707/400/0.8	226/1.6	2019	39
Sn/C-PANI^b^	855/100/0.1	153/10	2020	40
Sn@2DLMG c^c^	539/500/0.1	240/5	2021	19
C/Sn/HCNF^d^	610/200/0.2	317/2	2022	41
Sn@SiOC	547/200/1	538/5	2022	42
Sn@CNT	616.9/100/0.1	558/0.5	2023	43
Sn@C	542/100/0.1	535/0.5	2024	44
Sn@C/CNT	949/150/0.1	341/10	This work	

a Polyaniline.

b 1,2,4,5-Benzene-tetracarboxylic acid.

c Two dimensional laminar matrix of graphene composites.

d Helical carbon fibers.

e Cycles.

Among these materials, Sn@C/CNT demonstrates exceptional cyclic performance, achieving a remarkable specific capacity of 949 mA h g^−1^ at a current rate of 0.1 A g^−1^, even after 150 cycles. It also maintains an impressive specific capacity of 341 mA h g^−1^ at a highest current rate of 10 A g^−1^.

In comparison, some significant anode materials reported in the literature include the excellent monometallic anode material of Sn/C-PANI, which reaches a specific capacity of only 855 mA h g^−1^ at a current rate of 0.1 A g^−1^ after 100 cycles, and retains a specific capacity of 153 mA h g^−1^ at a 10 A g^−1^ rate. Additionally, the energetic bimetallic anode material Sn/MoS_2_/C shows a maximum specific capacity of 625 mA h g^−1^ at 1 A g^−1^ after 500 cycles, maintaining a specific capacity of 630 mA h g^−1^ at a current rate of 2 A g^−1^ for rate performance.

These results indicate that the synthesis method for the new Sn@C/CNT anode material has successfully achieved the goal of developing new Sn-based anode materials for LIBs by incorporating one-dimensional MWCNTs into the *in situ* carbon matrix.

### 3.3 Lithium ion storage and diffusion-controlled performances

**Fig. 7:**
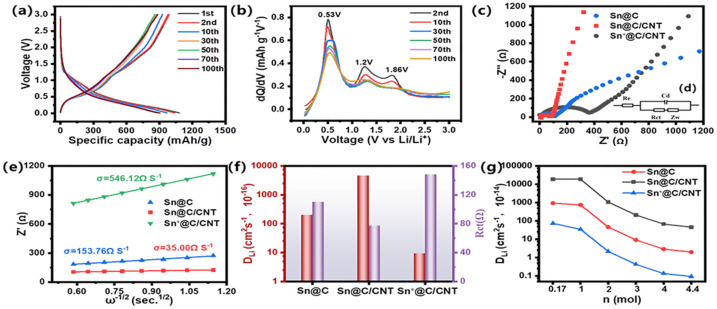
Lithium storage and diffusion-controlled dynamics analysis: (a) galvanostatic charge–discharge curves of Sn@C/CNT at 0.1 A g^−1^, (b) differential capacity curves (dQ/dV *vs.* voltage) of Sn@C/CNT at 0.1 A g^−1^; (c) electrochemical impedance spectroscopes; (d) (inset) equivalent circuit, (e) profiles of the real part of impedance (*Z*′) *vs. ω*^−1/2^, (f) profiles of *D*_Li^+^_, *R*_ct_*vs. ω*_w,_ and (g) lithium diffusion coefficients for three Sn-based anode materials based on reversible charge/discharge electro-chemical reaction of both SnLi*x* alloys and C_6_Li composites at different charge transfer numbers.

Fig. 7 discloses the dynamic behavior of three Sn-based anode materials in Lithium storage and interior diffusion behavior. As shown in Fig. 7(a), galvanostatic charge–discharge curves of Sn@C/CNT were tested at a current density of 0.1 A g^−1^, operating between 0.01 and 3.0 V. The initial charge and discharge capacities were approximately 1080 mA h g^−1^ and 988 mA h g^−1^, respectively, while the second cycle yielded 1037 mA h g^−1^ for charge and 978 mA h g^−1^ for discharge. Unlike the extremely low coulombic efficiency observed in SnO_*x*_ (1 ≤ *x*≤ 2) during the initial cycles,^45^ the coulombic efficiencies of the Sn-based anode material were notably high, reaching 91.5% and 94.2% in the first two cycles. This indicates that the alloying reaction provides a nearly reversible specific capacity, as outlined in Scheme 1, and suggests that minimal amounts of tin oxide impurities are present in the Sn@C/CNT composite. By the 30th cycle, stable charge and discharge capacities of 754.40 mA h g^−1^ and 743.37 mA h g^−1^ were achieved, with coulombic efficiencies remaining no less than 98.5%. This stability can primarily be attributed to the inevitable formation of a SEI film on the electrode surface. Fig. 7(b) further indicates the lithium storage performance originating from different reversible alloy reactions between Sn and Li. Three distinct extremes of differential capacity: a strong current peak around 0.53 V, a mediate current peak around 1.20 V, and a weak current peak around 1.83 V, respectively, represent different Sn–Li alloy species of Sn@C/CNT anode material during the redox reaction. The weak current peak around 1.83 V can be assigned to the irreversible desorption of Li^+^ from tin oxide due to its disappearance in subsequent cycling; the peaks around 0.53 V and 1.20 V repeated even after 100 charge/discharge cycles, can be associated with the formation of a series of Sn–Li alloy phases including Li_2_Sn_5_, Li_5_Sn_2_, Li_13_Sn_5_, Li_7_Sn_2_, and Li_22_Sn_5_ in the reversible desorption of lithium reactions.^46^ The mediate current peak of around 1.20 V finally indicates the formation of the Sn phase in the wholly delithiation.^11^

EIS analysis is widely used to evaluate the resistance of electrode materials and the diffusion of lithium ions within them.^45^ The equivalent circuit model used to fit the EIS spectrum is shown in the inset of Fig. 7(d). The series resistance (*R*_s_) is approximately 7.0 Ω, representing the resistance between the electrolyte and the anode materials, as illustrated in Fig. 7(c). This indicates the suitability of the selected electrolyte. In contrast, the charge transfer resistances (*R*_ct_) for Sn@C and Sn@C/CNT are 109.8 Ω and 148.1 Ω, respectively. The *R*_ct_ of 85.0 Ω for the Sn@C/CNT material shows the lowest charge transfer resistance during the electrochemical reaction, indicating superior performance. The Warburg factor (*σ*_w_), shown in Fig. 7(e), significantly influences the internal resistance related to lithium ion diffusion. This factor can be derived by calculating the slope of the fundamental part of resistance (Z′) *versus* the −1/2 exponent of angular frequency (*ω*^−1/2^) curves, as expressed in the following formula (1):^47^

1 *Z* = *R*_s_ + *R*_ct_ + *σ*_*ω*_*ω*^−1/2^

The diffusion-controlled lithium ion diffusion coefficient (*D*_Li^+^_) can subsequently be calculated using the Nyquist plot (2):^48^

2 *D*_Li^+^_ = *R*^2^*T*^2^/2*A*^2^*F*^4^*n*^4^*C*^2^*σ*_*W*_^2^

In this formula, *D*_Li^+^_ represents the lithium ion diffusion coefficient, *R* is the gas constant, *T* is the absolute temperature, *n* is the charge transfer number for each distinct charge/discharge reaction, *A* is the surface area of the electrode, *F* is Faraday's constant, and *C* is the lithium ion molar concentration for each anode material. According to the varied *σ*_w_ values (153.76, 35.00, and 546.12 Ω S^−1^) shown in Fig. 7(e) and (f) illustrates the *D*_Li^+^_ values for the three anode materials: 1.98 × 10^−14^ cm^2^ s^−1^ for Sn@C, 4.57 × 10^−13^ cm^2^ s^−1^ for Sn@C/CNT, and 9.25 × 10^−16^ cm^2^ s^−1^ for Sn*@C/CNT when considering the largest reversible alloy reaction (Li_4.4_Sn, *n* = 4.4). The detailed values of diffusion-controlled lithium-ion diffusion coefficients for these reversible redox reactions are shown in Table S1.†

The lowest *R*_ct_ and the highest *D*_Li^+^_ confirm that Sn@C/CNT material has a substantial advantage in electrochemical performance over the other two Sn-based materials. However, assuming that lithium ion diffusion efficiency stems solely from the Li_4.4_Sn alloy is an over-idealization.^49^Fig. 7(g) illustrates possible lithium ion diffusion pathways, incorporating the reversible charge/discharge chemical reactions from both C_6_Li composites^50^ and Li_*x*_Sn (where *x* = 1, 2, 3, or 4.4) alloys. The lithium ion diffusion coefficient for Sn@C/CNT from the C_6_Li composites is 1.9 × 10^−10^ cm^2^ s^−1^, while the coefficients from Li_*x*_Sn alloys (with *x* = 1, 2, 3, and 4) are 1.9 × 10^−10^, 1.07 × 10^−11^, 2.11 × 10^−12^, and 6.70 × 10^−13^ cm^2^ s^−1^, respectively. These diffusion coefficients decrease in order as the charge transfer number (*x*) increases during the reversible electrochemical reactions of the Li_*x*_Sn alloy, despite considering the counterbalancing effects of the lithium ion concentration and *R*_ct_ on *D*_Li^+^_. Similarly, Sn@C and Sn@C/CNT materials exhibit *D*_Li^+^_ values at least one hundred times lower across all stages of the Sn–Li alloy reaction, demonstrating the same trend in lithium diffusion coefficients. Given that the order of magnitude for the highest *D*_Li^+^_ is at least 10^−13^, it is unlikely that the total reversible specific capacity for the three Sn-based anode materials is solely derived from the higher lithium-rich alloy Li_4.4_Sn. Instead, a series of reversible electrochemical reactions likely indicates optimal diffusion-controlled dynamics, suggesting that up to five Sn–Li alloys can emerge during these charging and discharging cycles. Therefore, the normal charge transfer number is approximately three rather than 4.4 in these SnLi_*x*_ phases.^51^ Consequently, the optimal lithium diffusion coefficient is estimated to be in the range of 10^−9^ to 10^−10^. The promising anode material Sn@C/CNT shows adequate kinetics to sustain long-term cycling and achieves a higher specific capacity of 688 mA h g^−1^ at a current density of 1C, confirming its suitability.

In order to verify the analysis of diffusion-controlled lithium ion diffusion based on the Nyquist plot, Lithium ion coefficients of *D*_Li^+^_ based on the GITT curves for three anode materials were finished. The GITT *D*_Li^+^_ can be calculated by using the Weppner–Huggins plot:^52^3
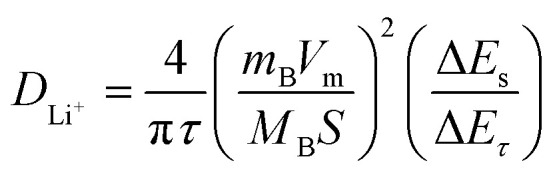
In this context, *m*_B_, *V*_m_, and *M*_B_ represent the mass, molar volume, and molar mass of active material, respectively. S represents the surface area of the electrode; Δ*E*_S_ refers to the change in open circuit voltage during the relaxation period, while Δ*E*_*τ*_ denotes the total change in cell voltage during pulse time.

**Fig. 8:**
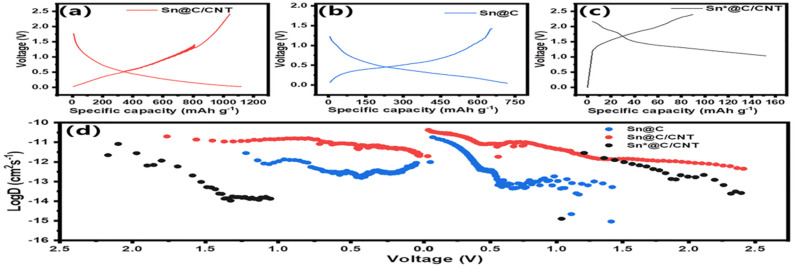
Analysis of lithium-ion storage and diffusion control kinetics: GITT charge and discharge curves (a) Sn@C/CNT (b) Sn@C and (c) Sn*@C/CNT, as well as (d) illustrates the variations in *D*_Li^+^_ across different discharge and charge voltages.

Fig. 8(a–c) demonstrate that both Sn@C/CNT and Sn@C anode materials exhibit more pronounced voltage hysteresis and higher GITT specific capacitance (1100 mA h g^−1^ for Sn@C/CNT and 700 mA h g^−1^ for Sn@C) compared to Sn*@C/CNT. This indicates that interior-controlled processes predominantly influence the lithium-ion dynamic behavior in the first two materials at a lower exchange current density of 0.1C. In contrast, surface-controlled lithium-ion dynamic behavior prevails for Sn*@C/CNT at the same current density.^53^Fig. 8(d) illustrates the variations in *D*_Li^+^_ across different discharge and charge voltages. When comparing the evolution of *D*_Li^+^_ for Sn@C/CNT, these values can be categorized into four distinct ranges: (a) *D*_Li^+^_ values in the order of magnitude of 10^−10^ to 10^−11^ cm^2^ s^−1^, occurring within a discharge voltage of 1.8 to 1.4 V and a charge voltage of 0 to 0.5 V; (b) *D*_Li^+^_ values around the order of magnitude of 10^−11^ cm^2^ s^−1^, within a discharge voltage of 1.4 to 0.75 V and a charge voltage of 0.5 to 0.75 V; (c) *D*_Li^+^_ values in the order of magnitude of 10^−12^ to 10^−13^ cm^2^ s^−1^, occurring within a discharge voltage of 0.75 to 0.5 V and a charge voltage of 0.75 to 1.5 V; (d) *D*_Li^+^_ values in the order of magnitude of 10^−14^ to 10^−15^ cm^2^ s^−1^, occurring within a discharge voltage of 0.5 to 0 V and a charge voltage of 1.5 to 2.5 V. The four components of *D*_Li^+^_ closely align with the analyses of various *D*_Li^+^_ variations corresponding to at least four electrochemical reactions. These include the intercalation/deintercalation lithium reaction of C_6_Li and the alloying reaction of Li_*x*_Sn (where *x* can be 1, 2, 3, or 4.4) for the Sn@C/CNT, as illustrated in Fig. 7.

Additionally, Fig. 8(d) also clearly shows that the Sn@C anode material exhibits lower *D*_Li^+^_ values across all four sections of *D*_Li^+^_ variation, and the Sn*@C/CNT anode material displays even lower *D*_Li^+^_ values, making it challenging to differentiate the four parts of the *D*_Li^+^_ distribution within similar discharging and charging voltages.

### 3.4 Lithium ion storage and capacity-controlled performance

**Fig. 9:**
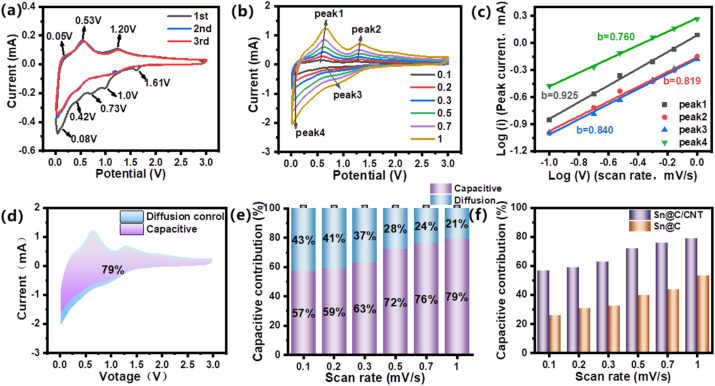
Capacity-controlled kinetics analyses: (a) CV curves for Sn@C/CNT anode material at a scan rate: 0.1 mV s^−1^; (b) LCV curves for Sn@C/CNT anode material from a scan rate: 0.1 to 1 mV s^−1^; (c) relationship between log(*i*) *versus* log(*v*) for Sn@C/CNT anode material; (d) and (e) capacity-controlled contributions for Sn@C/CNT anode material from 0.1 to 1 mV s^−1^; (f) Capacity-controlled contributions between Sn@C and Sn@C/CNT anode materials from 0.1 to 1 mV s^−1^.

To explore the capacity-controlled diffusion behavior of three Sn-based anode materials, the truth of lithium ion's storage, diffusion, and contribution from the pseudo-capacitance were further investigated. Fig. 9(a) illustrates the initial three CV curves of the Sn@C/CNT electrode, recorded at a scan rate of 0.1 mV s^−1^ within a voltage range of 0.01 to 3.0 V (*vs.* Li^+^/Li). In the first cathodic scan, two irreversible reduction peaks are observed around 1.17 V and 1.60 V. These peaks are attributed to the formation of metallic tin (Sn) and lithium oxide (Li_2_O) from SnO impurities, as indicated in Scheme 1(1) where *x* = 1, and the emergence of a SEI layer on the electrode surface.^54^ Additionally, reduction peaks at 0.42 V, 0.75 V, and 1.0 V are associated with the alloying process between lithium and tin, as depicted in Scheme 1(2) (1 ≤ *x* ≤ 4.4). During the subsequent anodic scan, two broad oxidation peaks at 0.53 V and 1.20 V confirm the presence of a reversible delithiation reaction in the Li_*x*_Sn alloy.^54^ Notably, the CV curves of Sn@C/CNT display a sharp reduction peak around 0.08 V and a prominent oxidation peak at 0.05 V. These peaks correspond to the reversible intercalation and delithiation of lithium ions into the carbon matrix, expressed as (C + *x*Li + *x*e ↔ Li_*x*_C).^54^ This coexistence of reversible delithiation and lithiation within the Sn@C/CNT material demonstrates a remarkable reinforcing effect that enhances lithium storage capacity. LCV curves of the electrode materials disclose the relationship between peak current (*i*) and scan rate (*v*). This power–law relationship can be expressed as the formula (4):^51^

4 log(*i*) = *b *log(*v*) + log* *(*a*)

The slope *b* in formula (4) is a adjustable parameter which be used to analyze the different diffusion mechanism of lithium ion in electrode material. Typically, when *b* = 0.5, the principal contribution derives from the diffusion-controlled process. As the *b*-value approaches 1.0, the capacity-controlled process dominates.^51^ As shown in Fig. 9(b), LCV curves of the Sn@C/CNT electrode, reveals the variety of summit currents at different scan rates from 0.1 to 1.0 mV s^−1^. Similar to two broad oxidation current peaks at 0.53 and 1.20 V in the anodic scans, two reduction peaks at 0.08 and 1.0 V in the cathodic scans were also chose to calculate the adjustable parameter *b* through plotting method based on the formula (5). Fig. 9(c) shows the b-values of the four peaks are 0.925, 0.819, 0.840 and 0.760 responding to summit currents from peak 1 to 4, respectively. It means that a mixed lithium ion diffusion mechanism is dominated by diffusion-controlled and surface-controlled processes.^51^Fig. 9(d)–(e) quantitatively describes the their contribution rate of the capacity-controlled diffusion to the total diffusion of lithium ion.^55^ It indicates that the capacity-controlled diffusion win more and more proportion in the total diffusion control for Sn@C/CNT material when the scan rate increased from 0.1 to 1.0 mV s^−1^, and the utmost contribution rate can be up to 79% at scan rate of 1.0 mV s^−1^. In contrast with those of Sn@C/CNT, the capacitive contribution of Sn@C electrode is lower at each scan rate, and only has as lower as 53% at the same scan rate of 1.0 mV s^−1^ (Fig. 9(f)).

The capacity-controlled diffusion of lithium ion can be known as the pseudo-capacitance control, and be studied in advanced by Randles–Sevcik eqn (5), in which the equation is always used to resolve the interface catalytic activity of the catalyst.^56^

**Fig. 10:**
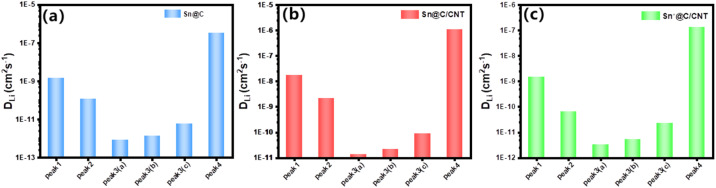
Lithium ion diffusion efficient based on the pseudo-capacitance: (a) *D*_Li^+^_ of Sn@C; (b) *D*_Li^+^_ of Sn@C/CNT anode material; (c) *D*_Li^+^_ of Sn*@C/CNT. Peak 1, 2, 3(a), 3(b), and 3(c) refer to a reversible Sn–Li alloy reaction between *y* = 2 and 3, *y* = 0 and 2, *y* = 0 and 4.4, *y* = 0 and 4, *y* = 0 and 3, respectively; and peak 4 refer to a reversible C_6_Li reaction between carbon matrix and Li. Tables S2–S7† provide detailed values of capacity-controlled lithium-ion diffusion coefficients for various reversible redox reactions.

5 *I*_p_ = 2.69 × 10^5^*n*^3/2^*A*(*D*_Li^+^_)^1/2^*ν*^1/2^Δ*C*_0_

wherein, *I*_p_ is the peak current (mA), *n* is the charges transfer molar number for every charge/discharge reaction, *A* is the surface area of the electrode (cm^2^), *D*_Li^+^_ is the Li^+^ diffusion coefficient (cm^2^ s^−1^), *ν* is the scan rate (mV s^−1^), Δ*C*_0_ is the change of molar concentration of Li^+^ before and after reaction. Based on the LCV curve for three Sn-based materials (see Fig. S10† for Sn@C, and Fig. S11† for Sn*@C/CNT), a series of *D*_Li^+^_ value revealing the diffusion behavior of three Sn-based material dominated by *I*_p_ was listed in Fig. 10(a)–(c).

Fig. 10(a) and (c) reveal that both Sn@C and Sn*@C/CNT all possess extraordinary lower delithiation–lithiation *D*_Li^+^_ than those of Sn@C/CNT as shown in Fig. 10(b). The lower adjustable parameter (*b* = 0.705, 0.592, 0.529 and 0.402 for Sn@C, see Fig. S10,† and *b* = 0.486, 0.445, 0.492 and 0.488 for Sn*@C/CNT, see Fig. S11†) further confirmed this conclusion.

The significant variations in the series of *D*_Li^+^_ values corresponding to different peak currents, as illustrated in Fig. 10(a)–(c), indicate distinct activation dynamics for each reversible reaction, whether it involves different Sn–Li alloys or the C–Li compound (C_6_Li). Furthermore, this suggests that the capacity-controlled diffusion performance of Sn@C and Sn*@C/CNT is considerably inferior compared to that of Sn @C/CNT.

The capacity-controlled lithium ion diffusion coefficients (*D*_Li^+^_) of Sn@C/CNT, as shown in Fig. 10(b), reveal significantly higher values in the context of reversible electrochemical reactions in Sn–Li alloys. These coefficients are one or two orders of magnitude greater than those calculated through Warburg factors (refer to Fig. 7(d)). Specifically, the average capacity-controlled *D*_Li^+^_ of 1.72 × 10^−8^ cm^2^ s^−1^, which corresponds to current peak 1 around 0.53 V, is nearly one hundred times higher than the diffusion-controlled *D*_Li^+^_ of 1.9 × 10^−10^ cm^2^ s^−1^ observed between Sn and SnLi (*x* = 1). Similarly, the average *D*_Li^+^_ of 2.15 × 10^−9^ cm^2^ s^−1^ associated with current peak 2 around 1.20 V is approximately two hundred times higher than the diffusion-controlled *D*_Li^+^_ of 1.07 × 10^−11^ cm^2^ s^−1^ found between Sn and SnLi (*x* = 2).

In contrast, the average *D*_Li^+^_ of 1.34 × 10^−11^ cm^2^ s^−1^, which corresponds to current peak 3(a) around 0.73 V, is only twenty times greater than the diffusion-controlled *D*_Li^+^_ of 6.70 × 10^−13^ cm^2^ s^−1^ between Sn and SnLi_4.4_ (*x* = 4.4). This indicates higher dynamic resistance and minimal formation of SnLi_4.4_ species. Similar trends can be observed in the average capacity-controlled *D*_Li^+^_ values for both Sn@C and Sn*@C/CNT anode materials (refer to Fig. 10(a) and (c)), compared to those of the two materials shown in Fig. 7(f).

These results align well with the increasing capacity-controlled diffusion behavior in Sn@C/CNT as the charge/recharge current density rises. This implies that reversible Sn–Li alloy reactions with lower charge transfer numbers (1 ≤ *y* ≤ 3) can generate significantly more pseudo-capacitance due to their enhanced dynamic activity.

For the C–Li compound (C_6_Li), its capacity-controlled *D*_Li^+^_ value corresponding to current peak 4 around 0.08 V is notably higher, reaching up to 1.08 × 10^−6^ cm^2^ s^−1^, compared to 1.90 × 10^−10^ cm^2^ s^−1^ obtained from EIS testing. This indicates that the carbon matrix has sufficient active sites on the surface of the anode material, enabling the generation of a substantial amount of pseudo-capacitance under large current densities.^57^ The higher *D*_Li^+^_ value results from the low resistance of the interfacial reaction between the carbon matrix and lithium ions. Additionally, the presence of carbon defects created by nearly 9.53 wt% nitrogen (N) and 9.52 wt% oxygen (O) in the *in situ* carbon composites significantly enhances Li^+^ transportation and contributes to the formation of more active sites.^58^

### 3.5 Mechanism of lithium storage and diffusion

The investigations summarized in Fig. 7 and 8 indicate that diffusion-controlled lithium-ion (*D*_Li^+^_) mechanisms arise from a minimum of four reversible electrochemical reaction pathways involving Sn–Li alloys. These reversible reactions inherently diminish the transmission efficiency of lithium ions, primarily due to the substantial phase transitions that occur within Sn–Li alloys.^12^ Furthermore, the active Sn-based sites within the carbon matrix necessitate that lithium ions surmount mass transfer resistance across effective ion transport lengths prior to reaching the surface of the anode material.^59^ Consequently, these diffusion-controlled dynamic behaviors are generally slower compared to the capacity-controlled diffusion observed in the surface redox reactions.^59^

**Fig. 11:**
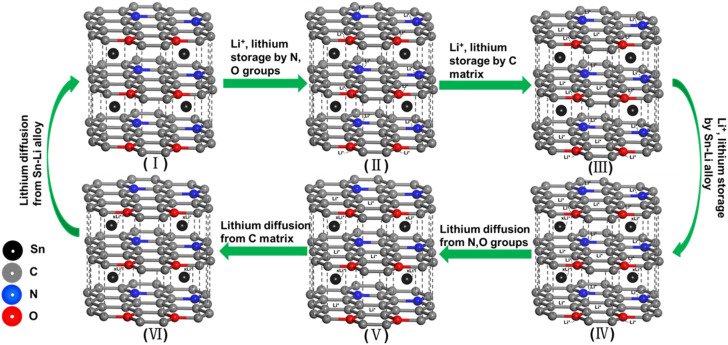
Storing and diffusing mechanism of lithium ion in Sn@C/CNT anode material: (I) graphite-like lamellar structure; (II) storage by N and O vacancy; (III) storage by carbon matrix; (IV) storage by Sn–Li alloy; (V) diffusion from N and O vacancy; (VI) diffusion from carbon matrix.

Therefore, Fig. 11 delineates a mixed storage and diffusion mechanisms of lithium ions that are influenced by diffusion-controlled and capacity-controlled behaviors in Sn@C/CNT anode material.

The structure of the obtained Sn@C/CNT anode material, as illustrated in Fig. 11(I), consists of a graphite-like layered nano-lamellar assembly interwoven with oxidized MWCNT fibers, within which tin nanoparticles are aligned. During the charging cycle, lithium ions are sequentially introduced into the material. First, they occupy the nitrogen (N) and oxygen (O) vacancies (Fig. 11(I)). They are trapped by the external active sites of the carbon matrix (Fig. 11(II)), and finally, they combine with the internal active tin nanoparticles (Fig. 11(III)). The third step of lithium insertion is slower than the first two due to unavoidable transmission resistance.

When lithium ions are extracted from the anode material, they follow the same sequence: they separate from the N and O vacancies (Fig. 11(IV)), are released from the carbon matrix (Fig. 11(V)), and then migrate from the inner tin nanoparticles (Fig. 11(VI)). Steps I, II, IV, and V can be categorized as capacity-controlled storage and diffusion behaviors since they involve minimal resistance in the external reactions. In contrast, steps III and VI are classified as diffusion-controlled storage and diffusion behaviors due to the apparent bulk resistance during the internal response.

This mixed mechanism of lithium-ion storage and diffusion ensures that the Sn@C/CNT anode material exhibits excellent long-term charge/discharge performance, achieving a capacity of up to 946 mA h g^−1^ at a low current rate of 0.1C. It also demonstrates an outstanding long-term cycling performance with a capacity of up to 688 mA h g^−1^ at a higher current density of 1 A g^−1^ (1C).

## 4. Conclusions

A three-dimensional reticulated Sn@C/CNT anode material was developed by annealing of a tin(II) complex with 2-methylimidazole at elevated temperatures and further interweaving with oxidized MWCNT fibers at a substantially lower annealing temperature. Comprehensive analyses, the unique topography for these Sn@C/CNT composites originate from their *in situ* Sn-based assemblies with the graphite-like nano-lamellar structure.

The Sn@C/CNT composites exhibited impressive long-term cycling spans at both the lower current density of 0.1C and the higher current density of 1C. The dynamic properties of the Sn@C/CNT anode materials are characterized by a higher diffusion-controlled lithium ion diffusion coefficient resulting from the internal reversible charge and discharge reactions of the Sn–Li alloy, as well as a higher capacity-controlled lithium ion diffusion coefficient originating from the pseudo-capacitance induced by the reversible charge and discharge reactions of both the Sn–Li alloy and the C_6_Li composites.

The topographical features of Sn@C/CNT are crucial for improving both the reversible specific capacity and the long-term cycling lifespan of the material. These features include nano-sized Sn active particles that measure as small as 2 to 3 nm, which are uniformly dispersed within a nano-lamellar carbon matrix, and the matrix consisting of graphite-like layers that are nearly 6 nm thick. Additionally, a comprehensive analytical approach to determining the mixed lithium-ion diffusion mechanism provides a deeper understanding of the electrochemical behavior of electrode materials during internal or interface reactions.

## Data availability

The data supporting this article have been included as part of the ESI.†

## Author contributions

Mengna Xie: conceptualization, methodology, writing–original draft preparation. Yongheng Zhou: methodology, data curation, Shuai Dong: visualization, writing–reviewing Fei Li: validation, writing–reviewing. Fenghua Zhang: writing–reviewing and editing. Wei Wei: visualization, writing–reviewing and editing. Jinhai Cui: supervision, visualization, writing–reviewing and editing.

## Conflicts of interest

We declare that there are no competing financial interests and/or personal relationships that could have appeared to influence the work reported in this paper.

## Supplementary Material

RA-015-D5RA02378E-s001

## References

[cit1] Liang M., Huang Y., Lin Y., Liang G., Huang C., Chen L., Li J., Feng Q., Lin C., Huang Z. (2021). Micro-nano structured VNb_9_O_25_ anode with superior electronic conductivity for high-rate and long-life lithium storage. J. Mater. Sci. Technol..

[cit2] Lv T.-B., Dai Y. K., Tan L., Zhang J.-J., Zhao Z.-Q., Liao K.-M., Wang H.-Y., Deng S., Dai G.-P. (2024). Hybrid 3D Vertical Graphene Nanoflake and Aligned Carbon Nanotube Architectures for High-Energy-Density Lithium-Ion Batteries. ACS Appl. Nano Mater..

[cit3] Zheng X., Song Z., Zhang D., Du W., Miao L., Lv Y., Xie L., Gan L., Liu M. (2024). Rational design of a dual-gradient zincophilic-conductive interphase for dendrite-free zinc batteries. J. Mater. Chem. A.

[cit4] Yi Z., Han Q., Zan P., Wu Y., Cheng Y., Wang L. (2016). Sb nanoparticles encapsulated into porous carbon matrixes for high-performance lithium-ion battery anodes. J. Power Sources.

[cit5] Liao K.-M., Dai Y. K., Wang H.-Y., Deng S., Dai G.-P. (2025). 3D Graphene Nanoflake/Vertically Aligned Carbon Nanotube/CoAl Layered Double Oxide Composites for High-Performance Lithium-Ion Batteries. ACS Appl. Energy Mater..

[cit6] Zhang Y., Song Z., Huang Q., Lv Y., Gan L., Liu M. (2025). Multiple Protophilic Redox-Active Sites in Reticular Organic Skeletons for Superior Proton Storage. Angew. Chem., Int. Ed..

[cit7] Zhang D., Song Z., Miao L., Lv Y., Duan H., Li M., Gan L., Liu M. (2025). Single Exposed Zn (0002) Plane and Sustainable Zn-Oriented Growth Achieving Highly Reversible Zinc Metal Batteries. Angew. Chem., Int. Ed..

[cit8] Sun Q., Geng L., Wang L., Che T., Tian D., Xu L.-C., Zhao J., Zhong Y., Wang Y., Yang Y., Kang L. (2024). Atomically Engineered Encapsulation of SnS_2_ Nanoribbons by Single-Walled Carbon Nanotubes for High-Efficiency Lithium Storage. Nano Lett..

[cit9] Idota Y., Kubota T., Matsufuji A., Maekawa Y., Miyasaka T. (1997). Tin-Based Amorphous Oxide: A High-Capacity Lithium-Ion-Storage Material. Science.

[cit10] Liu H., Hu R., Sun W., Zeng M., Liu J., Yang L., Zhu M. (2013). Sn@SnO_x_/C nanocomposites prepared by oxygen plasma-assisted milling as cyclic durable anodes for lithium ion batteries. J. Power Sources.

[cit11] Courtney I. A., Dahn J. R. (1997). Electrochemical and in situ X-Ray diffraction studies of the reaction of lithium with Tin oxide composites. J. Electrochem. Soc..

[cit12] Nam H. G., Park J. Y., Yuk J. M. (2022). Phase transformation mechanism and stress evolution in Sn anode. Energy Storage Mater..

[cit13] Liang S., Cheng Y. J., Zhu J., Xia Y., Müller-Buschbaum P. (2020). A Chronicle Review of Nonsilicon (Sn, Sb, Ge)-Based Lithium/Sodium-Ion Battery Alloying Anodes. Small Methods.

[cit14] Ye X., Lin Z., Liang S., Huang X., Qiu X., Qiu Y., Liu X., Xie D., Deng H., Xiong X., Lin Z. (2019). Upcycling of Electroplating Sludge into Ultrafine Sn@C Nanorods with Highly Stable Lithium Storage Performance. Nano Lett..

[cit15] Wang Y., Wu M., Jiao Z. (2009). Sn@CNT and Sn@C@CNT nanostructures for superior reversible lithium ion storage. Chem. Mater..

[cit16] Zhou X., Yu L., Yu X. Y. (2016). Encapsulating Sn Nanoparticles in Amorphous Carbon Nanotubes for Enhanced Lithium Storage Properties. Adv. Energy Mater..

[cit17] Qin J., He C., Zhao N., Wang Z., Shi C., Liu E. Z., Li J. (2014). Graphene Networks Anchored with Sn@Graphene as Lithium Ion Battery Anode. ACS Nano.

[cit18] Zhu C., Zhang Y., Wu Z., Ma Z., Guo X., Guo F., Zhang J., Li Y. (2021). Closely packed Si@C and Sn@C nano-particles anchored by reduced graphene oxide sheet boosting anode performance of lithium ion batteries. J. Mater. Sci. Technol..

[cit19] Ding S., Cheng W., Zhang L., Du G., Hao X., Nie G., Xu B., Zhang M., Su Q., Serra C. A. (2021). Organic molecule confinement reaction for preparation of the Sn nanoparticles@graphene anode materials in Lithium-ion battery. J. Colloid Interface Sci..

[cit20] Fan B., Liu J., Xu Y., Tang Q., Zhang Y., Chen X., Hu A. (2021). A facile strategy towards high capacity and stable Sn anodes for Li-ion battery: Dual-confinement via Sn@SnO_2_ core-shell nanoparticles embedded in 3D graphitized porous carbon network. J. Alloys Compd..

[cit21] Hou X., Jiang H., Hu Y., Li Y., Huo J., Li C. (2013). In situ deposition of hierarchical architecture assembly from Sn-filled CNTs for lithium-ion batteries. ACS Appl. Mater. Interfaces.

[cit22] Zhu J., Wang D., Cao L. (2014). Ultrafast preparation of three-dimensional porous tin–graphene composites with superior lithium ion storage. J. Mater. Chem. A.

[cit23] Vasnin S. V., Cetrullo J., Geanangel R. A., Bernal I. (1990). Adducts of 1-vinylimidazole, 1-benzylimidazole, and 1,2,4-triazole with tin(II) chloride. Inorg. Chem..

[cit24] Islam N., Dihingia A., Manna P., Das T., Kalita J., Dekaboruah H. P., Saikia B. K. (2019). Environmental and toxicological assessment of nanodiamond-like materials derived from carbonaceous aerosols. Sci. Total Environ..

[cit25] Ma B., Luo J., Deng X., Wu Z., Luo Z., Wang X., Wang Y. (2018). Hollow Silicon–Tin Nanospheres Encapsulated by N-Doped Carbon as Anode Materials for Lithium-Ion Batteries. ACS Appl. Nano Mater..

[cit26] Hu R., Ouyang Y., Liang T., Wang H., Liu J., Chen J., Yang C., Yang L., Zhu M. (2017). Stabilizing the Nanostructure of SnO_2_ Anodes by Transition Metals: A Route to Achieve High Initial Coulombic Efficiency and Stable Capacities for Lithium Storage. Adv. Mater..

[cit27] Fuertes A. B., Sevilla M. (2015). Hierarchical Microporous/Mesoporous Carbon composites for High-Performance Supercapacitors. ACS Appl. Mater. Interfaces.

[cit28] Zhu Z., Wang S., Du J., Jin Q., Zhang T., Cheng F., Chen J. (2013). Ultrasmall Sn Nanoparticles Embedded in Nitrogen-Doped Porous Carbon As High-Performance Anode for Lithium-Ion Batteries. Nano Lett..

[cit29] Zhang J., Jin B., Song Y., Hao W., Huang J., Guo J., Huang T., Guo Z., Peng R. (2021). Series of AzTO-Based
Energetic Materials: Effect of Different π–π Stacking Modes on Their Thermal Stability and Sensitivity. Langmuir.

[cit30] Ozeiry F., Ramezanzadeh M., Ramezanzadeh B. (2022). Multi-walled CNT decoration by ZIF-8 nanoparticles: O-MWCNT@ZIF-8/epoxy interfacial, thermal–mechanical properties analysis via combined DFT-D computational/experimental approaches. J. Ind. Eng. Chem..

[cit31] Majidi R., Ramezanzadeh M., Ramezanzadeh B. (2023). Developing a dual-functional self-healing nanocomposite utilizing oxidized-multiwall carbon nanotube/highly-porous metal-organic framework (OCNT/ZIF-8) nano-hybrid. Appl. Mater. Today.

[cit32] Qiu H., Zhao L., Asif M., Huang X., Tang T., Li W., Zhang T., Shen T., Hou Y. (2020). SnO_2_ nanoparticles anchored on carbon foam as a freestanding anode for high performance potassium-ion batteries. Energy Environ. Sci..

[cit33] Guo W., Wang Z., Wang X. (2021). General Design Concept for Single-Atom Catalysts toward Heterogeneous Catalysis. Adv. Mater..

[cit34] Zhang Z., Chen Y., Hu C., Zuo C., Wang P., Chen W., Ao T. (2021). Efficient removal of tetracycline by a hierarchically porous ZIF-8 metal organic framework. Environ. Res..

[cit35] Ying H., Zhang S., Meng Z., Sun Z., Han W.-Q. (2017). Ultrasmall Sn nanodots embedded inside N-doped carbon microcages as high-performance lithium and sodium ion battery anodes. J. Mater. Chem. A.

[cit36] Piao H., McIntyre N. S. (2002). Adventitious carbon growth on aluminium and gold–aluminium alloy surfaces. Surf. Interface Anal..

[cit37] Li Q.-Y., Pan Q.-C., Yang G.-H., Lin X.-L., Yan Z.-X., Wang H.-Q., Huang Y.-G. (2015). Synthesis of Sn/MoS_2_/C composites as high-performance anodes for lithium-ion batteries. J. Mater. Chem. A.

[cit38] Liang K., Cheang T. Y., Wen T., Xie X., Zhou X., Zhao Z. W., Shen C. C., Jiang N., Xu A. W. (2016). Facile Preparation of Porous Mn2SnO4/Sn/C Composite Cubes as High Performance Anode Material for Lithium-Ion Batteries. J. Phys. Chem. C.

[cit39] Xia S.-B., Yao L.-F., Guo H., Shen X., Liu J.-M., Cheng F.-X., Liu J.-J. (2019). Li^+^ intercalation pseudocapacitance in Sn-based metal-organic framework for high capacity and ultra-stable Li ion storage. J. Power Sources.

[cit40] Li Y., Ou C., Zhu J., Liu Z., Yu J., Li W., Zhang H., Zhang Q., Guo Z. (2020). Ultrahigh and durable volumetric lithium/sodium storage enabled by a highly dense graphene-encapsulated nitrogen-doped carbon@Sn compact monolith. Nano Lett..

[cit41] Chen G., Jin Y., Su W., Li Y., Zhang W., Qing T. (2022). C/Sn deposition on a helical carbon nanofiber matrix as a high performance anode for lithium-ion batteries. New J. Chem..

[cit42] Eun Wang S., Park J.-S., Ji Kim M., Chan Kang Y., Soo Jung D. (2022). One-pot spray pyrolysis for core–shell structured Sn@SiOC anode nanocomposites that yield stable cycling in lithium-ion batteries. Appl. Surf. Sci..

[cit43] Sadatian Abkenar S. A., Borghei S. M., Monsefi M. (2023). Preparation and evaluation of an Efficient Si-CNT Anode Decorated with Sn for Lithium-Ion Batteries. J. Electron. Mater..

[cit44] Liu X., Fang Y., Shi C., Fu H., Yao S. (2024). Sn@C fiber prepared by electrospinning as anode materials for lithium-ion batteries. J. Mater. Sci.: Mater. Electron..

[cit45] Wang C., Huang J., Li J., Wang H., Kang S., Cao L., Kajiyoshi K. (2021). Improving surface activity of TiO_2_ by introducing Co sources to enhance its ability to capture polysulfides. Ceram. Int..

[cit46] Cheong J. Y., Kim C., Jung J.-W., Yoon K. R., Kim I.-D. (2018). Porous SnO_2_-CuO nanotubes for highly reversible lithium storage. J. Power Sources.

[cit47] Zhu R., Wang Z., Hu X., Liu X., Wang H. (2021). Silicon in Hollow Carbon Nanospheres Assembled Microspheres Cross-linked with N-doped Carbon Fibers toward a Binder Free, High Performance, and Flexible Anode for Lithium-Ion Batteries. Adv. Funct. Mater..

[cit48] Ivanishchev A. V., Ushakov A. V., Ivanishcheva I. A., Churikov A. V., Mironov A. V., Fedotov S. S., Khasanova N. R., Antipov E. V. (2017). Structural and electrochemical study of fast Li diffusion in Li_3_V_2_(PO_4_)_3_-based electrode material. Electrochim. Acta.

[cit49] Wu Q., Shao Q., Li Q., Duan Q., Li Y., Wang H.-g. (2018). Dual Carbon-Confined SnO_2_ Hollow Nanospheres Enabling High Performance for the Reversible Storage of Alkali Metal Ions. ACS Appl. Mater. Interfaces.

[cit50] Dahn J. R., Zheng T., Liu Y. (1995). Mechanisms for Lithium Insertion in Carbonaceous Materials. Science.

[cit51] Wang T., Legut D., Fan Y., Qin J., Li X., Zhang Q. (2020). Building Fast Diffusion Channel by Constructing Metal Sulfide/Metal Selenide Heterostructures for High-Performance Sodium Ion Batteries Anode. Nano Lett..

[cit52] Weppner W., Huggins R. A. (1978). Electrochemical Methods for Determining Kinetic Properties of Solids. Annu. Rev. Mater. Res..

[cit53] Heubner C., Schneider M., Michaelis A. (2016). SoC dependent kinetic parameters of insertion electrodes from staircase — GITT. J. Electroanal. Chem..

[cit54] Cheng Y., Yi Z., Wang C., Wu Y., Wang L. (2017). Controllable fabrication of C/Sn and C/SnO/Sn composites as anode materials for high-performance lithium-ion batteries. Chem. Eng. J..

[cit55] Yao W., Wu S., Zhan L. (2019). Two-dimensional porous carbon-coated sandwich-like mesoporous SnO_2_/graphene/mesoporous SnO_2_ composites towards high-rate and long cycle life lithium-ion batteries. Chem. Eng. J..

[cit56] Abdi Z., Vandichel M., Sologubenko A. S., Willinger M.-G., Shen J.-R., Allakhverdiev S. I., Najafpour M. M. (2021). The importance of identifying the true catalyst when using Randles-Sevcik equation to calculate turnover frequency. Int. J. Hydrogen Energy.

[cit57] Liu S., Liu K., Chen K., Fu J., Li H., An P., Li H., Jia C., Xie H., Liu H., Hu J., Pan H., Zheng X., Liu X., Wang X., Liu M. (2020). Tailoring the structure of supported δ-MnO_2_ composites to raise pseudocapacitance by surface-modified carbon cloth. J. Power Sources.

[cit58] Hu Y.-r., Dong X.-l., Hou L., Zhuang H.-k., Li W.-c. (2021). Electrochemical oxidation of 2D B, N-codoped carbon composites to improve their pseudo-capacitance. New Carbon Mater..

[cit59] Song G., Huang X., Feng H., Zuo Z., Li J., Tang D., Wei Q., Mei B.-A. (2023). Physical interpretations of diffusion-controlled intercalation and surface-redox charge storage behaviors. Energy Storage Mater..

