# Supplementary material for: Effective anode materials for in situ Sn@C nano-lamellar assembly with doped nanotubes in lithium-ion batteries
Source: RSC Adv. 2025 Jun 6;15(24):19176–91. doi: 10.1039/d5ra02378e (PMC12142737; doi:10.1039/d5ra02378e)
Supplement: RA-015-D5RA02378E-s001 [file RA-015-D5RA02378E-s001.pdf]

Supplementary Information (SI) for RSC Advances.
This journal is © The Royal Society of Chemistry 2025

Supplementary

Effective anode materials for in-situ Sn/C nanosheets with the doped nanotubes in lithium-ion batteries

Meng-na Xie[ab] ,Yong-heng Zhou[c] ,shuai Dong[b], Fei Li[a], Feng-hua Zhang[*a], Wei Wei[*b] and Jin-hai Cui[*b]

[a] School of Petrochemical engineering, Liaoning Petrochemical University, Fushun 113001, P. R. China

[b] Henan Engineering Center of New Energy Battery Materials, School of Chemistry and Chemical Engineering, Shangqiu Normal University, Shangqiu 476000, P. R. China

[c] Kaifeng University, school of material and chemical engineering, Kaifeng 475000, P. R. China.

[*] Corresponding author. School of Chemistry and Chemical Engineering, Shangqiu Normal University, Shangqiu 476000, P. R. China

E-mail addresses: cuijinghai535@gmail.com (Jinhai Cui), weiweizzuli@163.com (Wei Wei)

1. Computational details

1.1 diffusion-controlled lithium ion diffusion coefficients

The diffusion-controlled lithium ion diffusion coefficient (D_{Li+}) can subsequently be calculated using the formula (2) :

$$D_{Li^+} = R^2T^2 \Big/ 2A^2F^4n^4C^2\sigma_W^2 \qquad (2)$$

In this formula, D_{Li+} represents the lithium ion diffusion coefficient, R is the gas constant, T is the absolute temperature, n is the charge transfer number for each distinct charge/discharge reaction, A is the surface area of the electrode, F is Faraday's constant, and C is the lithium ion molar concentration for each anode material. The calculations are summarized in Table S1.

Table S1 diffusion-controlled lithium ion diffusion coefficient of three anodes at different redoxs

Anodes	parameters							D_{Li+}
	R J/mol·K	T (K)	A (cm^2)	F C/mol	n	C (mol/mL)	σ_w $\Omega \cdot S^{1/2}$	cm^2/s
Sn@C	8.314	298.15	1	96500	0.17	0.014	153.76	9.36E-12
Sn@C/CNT	8.314	298.15	1	96500	0.17	0.014	35	1.90E-10
Sn*@C/CNT	8.314	298.15	1	96500	0.17	0.014	546.12	7.42E-13
Sn@C	8.314	298.15	1	96500	1.0	0.0004	153.76	7.45E-12
Sn@C/CNT	8.314	298.15	1	96500	1.0	0.0004	35	1.9E-10
Sn*@C/CNT	8.314	298.15	1	96500	1.0	0.0004	546.12	3.47E-13
Sn@C	8.314	298.15	1	96500	2.0	0.0004	153.76	4.65E-13
Sn@C/CNT	8.314	298.15	1	96500	2.0	0.0004	35	1.07E-11
Sn*@C/CNT	8.314	298.15	1	96500	2.0	0.0004	546.12	2.16E-14
Sn@C	8.314	298.15	1	96500	3.0	0.0004	153.76	9.18E-14
Sn@C/CNT	8.314	298.15	1	96500	3.0	0.0004	35	2.11E-12
Sn*@C/CNT	8.314	298.15	1	96500	3.0	0.0004	546.12	4.28E-15
Sn@C	8.314	298.15	1	96500	4.0	0.0004	153.76	2.91E-14
Sn@C/CNT	8.314	298.15	1	96500	4.0	0.0004	35	6.7E-13
Sn*@C/CNT	8.314	298.15	1	96500	4.0	0.0004	546.12	3.5E-15
Sn@C	8.314	298.15	1	96500	4.4	0.0004	153.76	1.98E-14
Sn@C/CNT	8.314	298.15	1	96500	4.4	0.0004	35.00	4.57E-13
Sn*@C/CNT	8.314	298.15	1	96500	4.4	0.0004	546.12	9.25E-16

1.2 capacity-controlled lithium ion diffusion coefficients

The capacity-controlled diffusion of lithium ion can be known as the pseudo-capacitance control, and be studied in advanced by Randles-Sevcik equation (4), in which the equation is always used to resolve the interface catalytic activity of the catalyst.

$$I_p=2.69\times10^5\times n^3/2A\times(D_{Li+})^{1/2}\times\nu^{1/2}\times\Delta C_0 \qquad (4)$$

Wherein, Ip is the peak current (mA) in LCV testing, n is the charges transfer molar number for every charge/discharge reaction, A is the surface area of the electrode (cm^2), D_{Li+} is the Li^+ diffusion coefficient (cm^2/s), ν is the scan rate (mv/s), ΔC_0 is the change of molar concentration of Li^+ before and after reaction. The peak currents of different reversible Sn-Li alloy reaction were annotated as Ip_1, Ip_2, $Ip_{3(a–c)}$ and Ip_4. The calculations are summarized in Table S2–7.

§ peak 1, 2, 3(a), 3(b), and 3(c) refer to a reversible Sn-Li alloy reaction between y=2 and 3, y= 0 and 2, y= 0 and 4.4, y= 0 and 4, x= 0 and 3, respectively; and peak 4 refer to a reversible C_6Li reaction between carbon matrix and Li.

Table S2 capacity-lithium ion diffusion coefficient of three anodes based on the redox of

$Li_2Sn+Li^++e^-\leftrightarrow Li_3Sn$

$(Sn+yLi^++ye^-\leftrightarrow Li_ySn\ (0\leq y\leq 4.4))$

Anodes	parameters					D_{Li+}
	V (Voltage)	Ip_1 mA	$\triangle C_0$ mol/mL	n	A cm^2	cm^2/s
Sn@C	1	0.173	0.0008	1	1	6.48E-10
	0.7	0.1648	0.0008	1	1	8.40E-10
	0.5	0.1583	0.0008	1	1	1.09E-09
	0.3	0.1442	0.0008	1	1	1.50E-09
	0.2	0.1336	0.0008	1	1	1.93E-09
	0.1	0.1189	0.0008	1	1	3.06E-09
Sn@C/CNT	1	1.2524	0.0008	1	1	3.40E-08
	0.7	0.8576	0.0008	1	1	2.28E-08
	0.5	0.6161	0.0008	1	1	1.64E-08
	0.3	0.4481	0.0008	1	1	1.45E-08
	0.2	0.2969	0.0008	1	1	9.55E-09
	0.1	0.1667	0.0008	1	1	6.02E-09
Sn*@C/CNT	1	0.2138	0.0008	1	1	9.90E-10
	0.7	0.2	0.0008	1	1	1.24E-09
	0.5	0.1947	0.0008	1	1	1.64E-09
	0.3	0.1816	0.0008	1	1	2.38E-09
	0.2	0.127	0.0008	1	1	1.75E-09
	0.1	0.0729	0.0008	1	1	1.15E-09

Table S3 capacity-lithium ion diffusion coefficient of three anodes based on the redox of

$$Sn+2Li^{+}+2e^{-}\leftrightarrow Li_2Sn$$

$$(Sn+2Li^{+}+2e^{-}\leftrightarrow Li_ySn\ (0\leq y\leq 4.4))$$

Anodes	parameters					D_{Li+}
	V (Voltage)	I_{p2} mA	$\triangle C_0$ mol/mL	n	A cm2	cm^2/s
Sn@C	1	0.2141	0.0008	2	1	1.24E-10
	0.7	0.1871	0.0008	2	1	1.35E-10
	0.5	0.1542	0.0008	2	1	1.29E-10
	0.3	0.1189	0.0008	2	1	1.28E-10
	0.2	0.09	0.0008	2	1	1.10E-10
	0.1	0.055	0.0008	2	1	8.19E-11
Sn@C/CNT	1	1.2524	0.0008	2	1	4.25E-09
	0.7	0.8576	0.0008	2	1	2.84E-09
	0.5	0.6161	0.0008	2	1	2.06E-09
	0.3	0.4481	0.0008	2	1	1.81E-09
	0.2	0.2969	0.0008	2	1	1.19E-09
	0.1	0.1667	0.0008	2	1	7.52E-10
Sn*@C/CNT	1	0.175	0.0008	2	1	8.29E-11
	0.7	0.1302	0.0008	2	1	6.56E-11
	0.5	0.1041	0.0008	2	1	5.87E-11
	0.3	0.08	0.0008	2	1	5.78E-11
	0.2	0.07	0.0008	2	1	6.63E-11
	0.1	0.05	0.0008	2	1	6.77E-11

Table S4 capacity-lithium ion diffusion coefficient of three anodes based on the redox of

$$Sn+4.4Li^{+}+4.4e^{-}\leftrightarrow Li_{4.4}Sn$$

$$(Sn+yLi^{+}+ye^{-}\leftrightarrow Li_{y}Sn\ (0\leq y\leq 4.4))$$

Anodes	parameters					D_{Li+}
	V (Voltage)	$I_{p3(a)}$ mA	$\triangle C_0$ mol/mL	n	A cm^2	cm^2/s
Sn@C	1	0.1174	0.00176	4.4	1	7.24E-13
	0.7	0.099	0.00176	4.4	1	7.36E-13
	0.5	0.0868	0.00176	4.4	1	7.92E-13
	0.3	0.068	0.00176	4.4	1	8.10E-13
	0.2	0.058	0.00176	4.4	1	8.84E-13
	0.1	0.05	0.00176	4.4	1	1.31E-12
Sn@C/CNT	1	0.6816	0.00176	4.4	1	2.44E-11
	0.7	0.5073	0.00176	4.4	1	1.93E-11
	0.5	0.3708	0.00176	4.4	1	1.44E-11
	0.3	0.249	0.00176	4.4	1	1.09E-11
	0.2	0.1671	0.00176	4.4	1	7.33E-12
	0.1	0.0894	0.00176	4.4	1	4.20E-12
Sn*@C/CNT	1	0.255	0.00176	4.4	1	3.42E-12
	0.7	0.205	0.00176	4.4	1	3.15E-12
	0.5	0.1792	0.00176	4.4	1	3.37E-12
	0.3	0.147	0.00176	4.4	1	3.78E-12
	0.2	0.1186	0.00176	4.4	1	3.69E-12
	0.1	0.0728	0.00176	4.4	1	2.78E-12

Table S5 capacity-lithium ion diffusion coefficient of three anodes based on the redox of

$$Sn+4\ Li^{+}+4\ e^{-}\leftrightarrow Li_4Sn$$

$$(Sn+yLi^{+}+ye^{-}\leftrightarrow Li_ySn\ (0\leq y\leq 4.4))$$

Anodes	parameters					D_{Li+}
	V (Voltage)	$I_{p3(b)}$ mA	$\triangle C_0$ mol/mL	n	A cm^2	cm^2/s
Sn@C	1	0.1174	0.0016	4	1	1.17E-12
	0.7	0.099	0.0016	4	1	1.19E-12
	0.5	0.0868	0.0016	4	1	1.28E-12
	0.3	0.068	0.0016	4	1	1.30E-12
	0.2	0.058	0.0016	4	1	1.42E-12
	0.1	0.05	0.0016	4	1	2.12E-12
Sn@C/CNT	1	0.6816	0.0016	4	1	3.93E-11
	0.7	0.5073	0.0016	4	1	3.11E-11
	0.5	0.3708	0.0016	4	1	2.33E-11
	0.3	0.249	0.0016	4	1	1.75E-11
	0.2	0.1671	0.0016	4	1	1.18E-11
	0.1	0.0894	0.0016	4	1	6.76E-12
Sn*@C/CNT	1	0.255	0.0016	4	1	5.50E-12
	0.7	0.205	0.0016	4	1	5.08E-12
	0.5	0.1792	0.0016	4	1	5.43E-12
	0.3	0.147	0.0016	4	1	6.09E-12
	0.2	0.1186	0.0016	4	1	5.95E-12
	0.1	0.0728	0.0016	4	1	4.48E-12

Table S6 capacity-lithium ion diffusion coefficient of three anodes based on the redox of

$$Sn+3\ Li^{+}+3\ e^{-} \leftrightarrow Li_3Sn$$

$$(Sn+yLi^{+}+ye^{-} \leftrightarrow Li_ySn\ (0 \leq y \leq 4.4))$$

Anodes	parameters					D_{Li+}
	V (Voltage)	$I_{p3(c)}$ mA	$\triangle C_0$ mol/mL	n	A cm^2	cm^2/s
Sn@C	1	0.1174	0.0012	3	1	4.91E-12
	0.7	0.099	0.0012	3	1	4.99E-12
	0.5	0.0868	0.0012	3	1	5.37E-12
	0.3	0.068	0.0012	3	1	5.50E-12
	0.2	0.058	0.0012	3	1	6.00E-12
	0.1	0.05	0.0012	3	1	8.91E-12
Sn@C/CNT	1	0.6816	0.0012	3	1	1.66E-10
	0.7	0.5073	0.0012	3	1	1.31E-10
	0.5	0.3708	0.0012	3	1	9.80E-11
	0.3	0.249	0.0012	3	1	7.37E-11
	0.2	0.1671	0.0012	3	1	4.98E-11
	0.1	0.0894	0.0012	3	1	2.85E-11
Sn*@C/CNT	1	0.255	0.0012	3	1	2.32E-11
	0.7	0.205	0.0012	3	1	2.14E-11
	0.5	0.1792	0.0012	3	1	2.29E-11
	0.3	0.147	0.0012	3	1	2.57E-11
	0.2	0.1186	0.0012	3	1	2.51E-11
	0.1	0.0728	0.0012	3	1	1.89E-11

Table S7 capacity-lithium ion diffusion coefficient of three anodes based on the redox of

$$C+0.17\ Li^{+}+0.17\ e^{-} \leftrightarrow CLi_{0.17}$$

Anodes	parameters					D_{Li+}
	V (Voltage)	I_{p4} mA	$\triangle C_0$ mol/mL	n	A cm^2	cm^2/s
Sn@C	1	0.648	0.0025	0.17	1	2.01E-07
	0.7	0.624	0.0025	0.17	1	2.66E-07
	0.5	0.5154	0.0025	0.17	1	2.54E-07
	0.3	0.4866	0.0025	0.17	1	3.78E-07
	0.2	0.456	0.0025	0.17	1	4.98E-07
	0.1	0.3132	0.0025	0.17	1	4.70E-07
Sn@C/CNT	1	1.828	0.0025	0.17	1	1.60E-06
	0.7	1.456	0.0025	0.17	1	1.50E-06
	0.5	1.154	0.0025	0.17	1	1.28E-06
	0.3	0.7593	0.0025	0.17	1	9.20E-07
	0.2	0.53	0.0025	0.17	1	6.72E-07
	0.1	0.341	0.0025	0.17	1	5.57E-07
Sn*@C/CNT	1	0.5532	0.0025	0.17	1	1.47E-07
	0.7	0.4692	0.0025	0.17	1	1.51E-07
	0.5	0.3856	0.0025	0.17	1	1.42E-07
	0.3	0.3179	0.0025	0.17	1	1.61E-07
	0.2	0.2246	0.0025	0.17	1	1.20E-07
	0.1	0.1246	0.0025	0.17	1	7.43E-08

2. XRD patterns for Sn-C composites

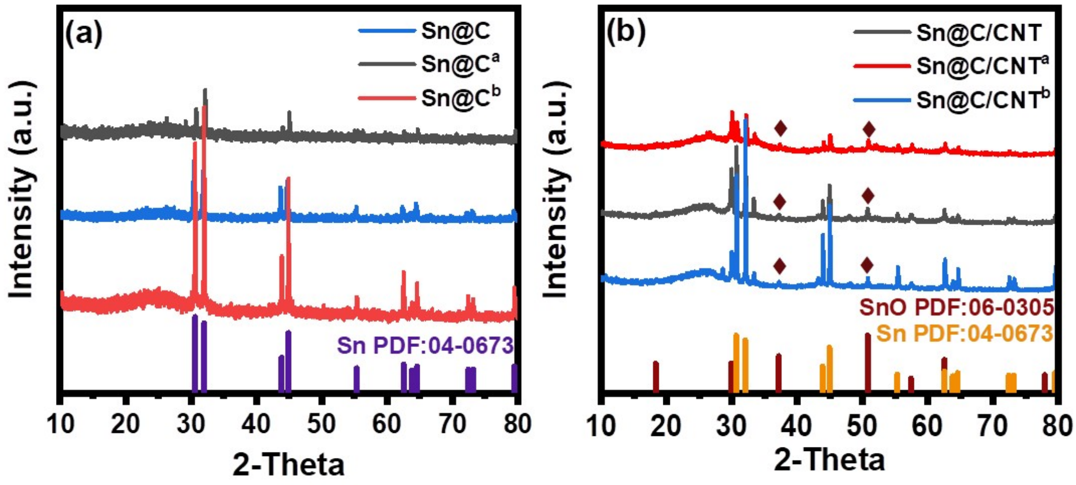

Fig. S1 (a) XRD patterns for Sn@C[a], Sn@C, and Sn@C[b] annealed at 700 °C, 800 °C and 900 °C retrospectively; (b) XRD patterns for Sn@C/CNT[a], Sn@C/CNT, and Sn@C/CNT[b] annealed at 700 °C, 800 °C and 900 °C retrospectively

Composites of Sn@C[a], Sn@C, and Sn@C[b] that were annealed at temperatures of 700

°C, 800 °C, or 900 °C show increased crystallization as the annealing temperature rises, as illustrated in Fig. 1S(a). Higher annealing temperatures facilitate the formation of the Sn phase with larger particle sizes, which leads to more pronounced bulk expansion during the redox reactions of Sn-Li alloys. In contrast, lower annealing temperatures result in a higher Sn content in the Sn@C complex, which ultimately impairs cycling performance. Therefore, an optimal annealing temperature of 800 °C is selected, and this temperature is also applied to the Sn@C/CNT^{a}, Sn@C/CNT, and Sn@C/CNT^{b} composites. Additionally, Fig. 1S(b) reveals the presence of SnO impurities in the XRD patterns of both Sn@C/CNT^{a} and Sn@C/CNT^{b} anode materials, further indicating an oxidation reaction between the oxidized MWCNT and the active Sn phase.

3. SEM patterns for Sn-C composites

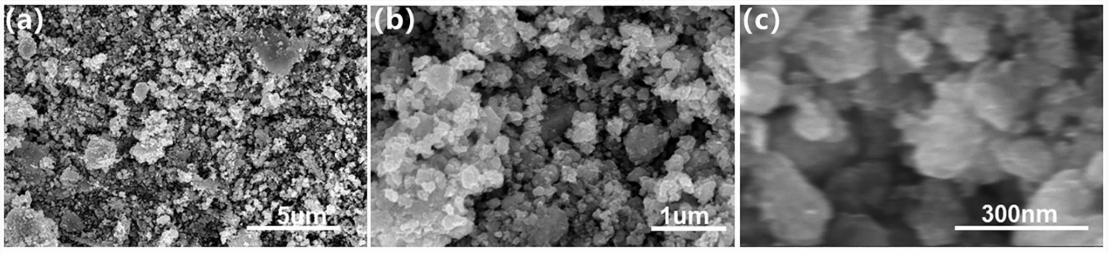

Fig. S2 (a, b and c) SEM patterns for commercial Sn*@C/CNT at 800 °C

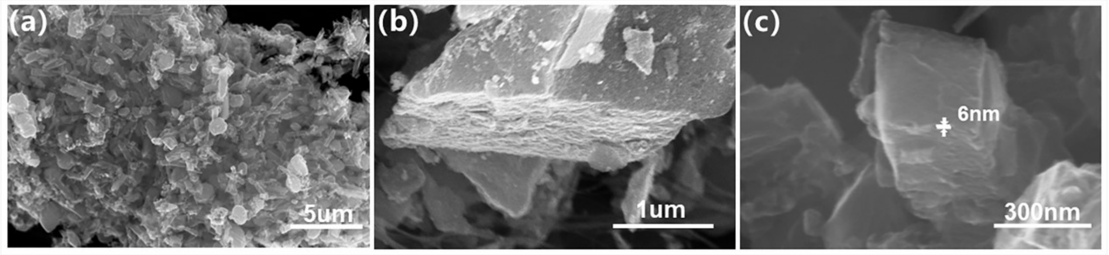

Fig. S3 (a, b and c) SEM patterns for commercial Sn@C at 800 °C

Figure S2 shows the topographies of commercial Sn@C particles that were annealed under the same conditions as those of the Sn@C samples. The analysis revealed spherical nano-sized Sn particles embedded in a disordered carbon matrix, without any layered carbon structure present. In contrast, Figure S3 illustrates that the Sn@C particles exhibit graphite-like, layered micro-lamellar assemblies. According to measurements, this thin graphite-like film has an average thickness of just 6 nm.

4. EDS mappings for Sn-C composites

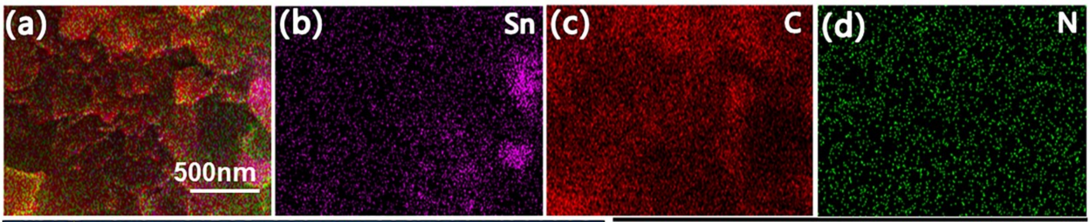

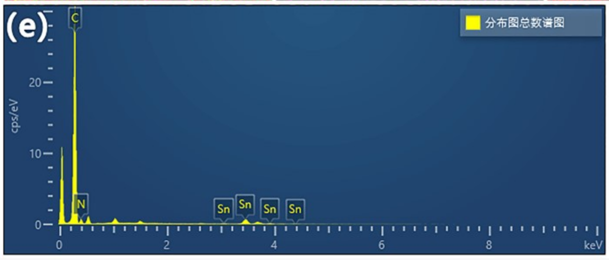

element	apparent concentration	Wt%
C	134.42	69.10
N	30.14	12.27
Sn	25.39	18.63
gross:		100.00

Fig. S4 (a–e) EDS mappings for Sn@C annealed at 800 °C

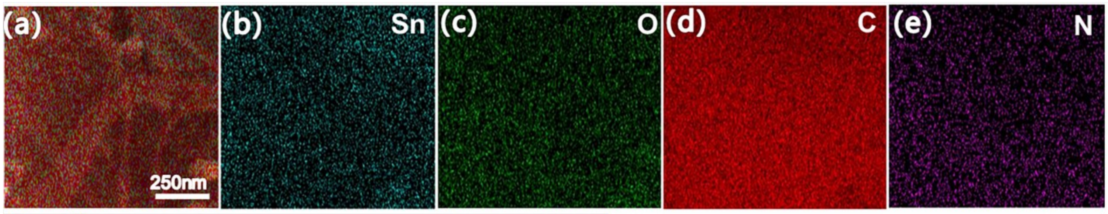

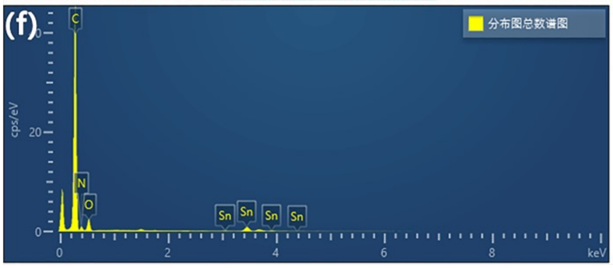

element	apparent concentration	Wt%
C	151.17	65.89
N	29.38	9.53
O	20.76	9.52
Sn	24.84	15.06
gross:		100.00

Fig. S5 (a–f) EDS mappings for Sn@C/CNT annealed at 800 °C

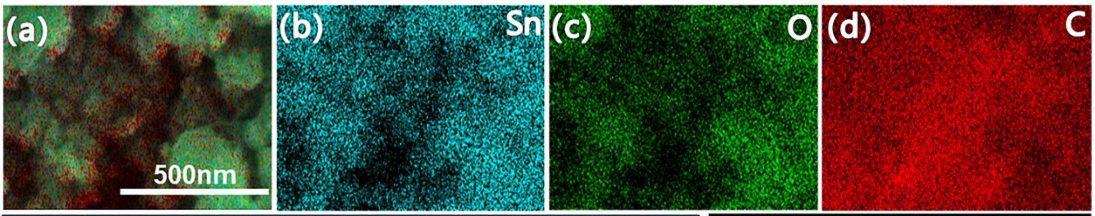

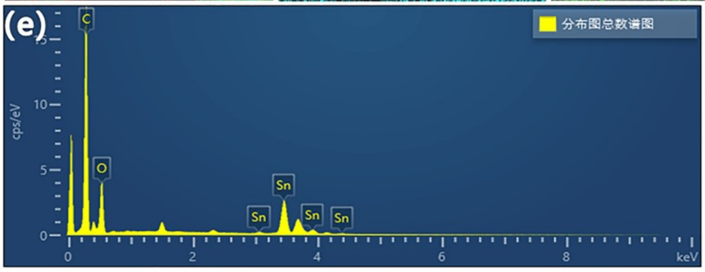

element	apparent concentrati on	Wt%
C	69.84	33.02
O	42.25	17.04
Sn	94.27	49.94
gross:		100.00

Fig. S6 (a–e) EDS mappings for Sn@C/CNT* annealed at 800 °C

EDS observations and corresponding mappings of the Sn@C particles reveals the presence of Nitrogen (N) in the Sn@C material as shown in ig. S4, which can be reasonably attributed to the

incomplete oxidation of amino groups in the presence of Sn^{2+} . The EDS analysis and mappings of Sn@C/CNT particles shown in Fig. S5, indicate the presence of oxygen (O) alongside the Tin (Sn) and nitrogen (N); this O contributes to the tiny SnO impurities observed in the composite. Both the Sn and N elements in the Sn@C particle, as well as the Sn, N, and O elements in the Sn@C/CNT particle, are uniformly distributed throughout their carbon-based matrix. In contrast, the elements in the Sn*@C/CNT particle are unevenly distributed as shown in Fig. S6.

5. XPS analysis for Sn-C composites

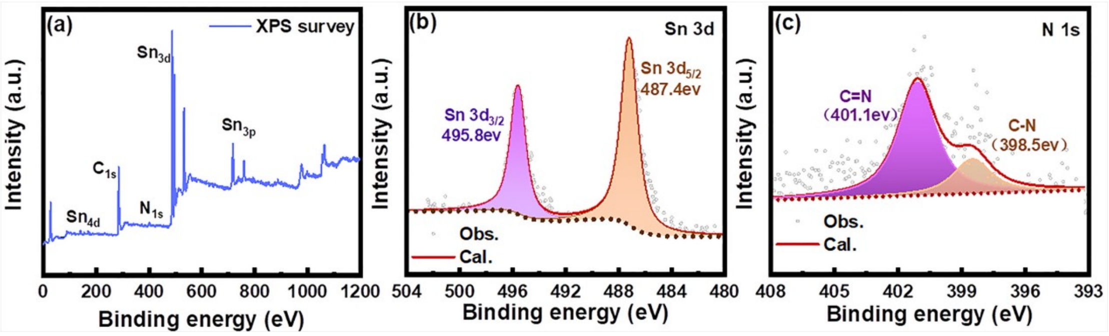

Fig. S7 (a–c) XPS Sn@C composite annealed at 800 °C

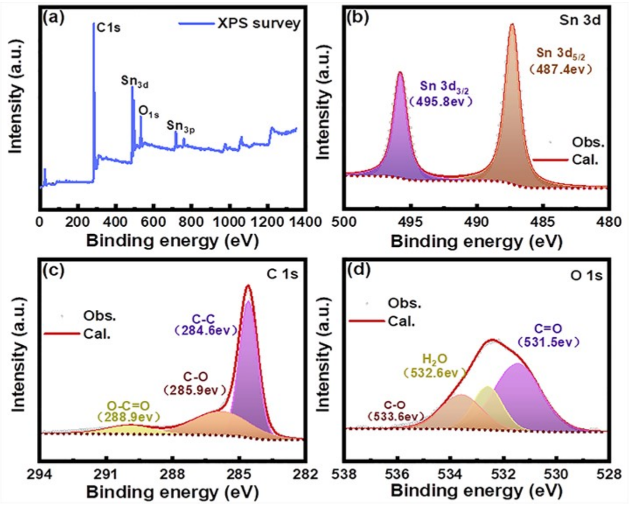

Fig. S8 (a–d) XPS of Sn@C/CNT*composite annealed at 800 °C

Unlike the XPS spectra of Sn@C/CNT, the Sn@C material only exhibited peaks for Sn, N, and C in Fig.S7. The C1s and N1s spectra for Sn@C showed BE peaks consistent with those observed in Sn@C/CNT. Two deconvoluted BE peaks, depicted in Fig. 5(f), are located at 284.6 eV, corresponding to the signature of graphite (sp^2 C=C) and amino groups, respectively. Additionally, the BE peak at 288.9 eV can be attributed to the unavoidable absorption of oxygen by the active carbon matrix of

Sn@C material. The XPS spectra of Sn*@C/CNT show in Fig.S8 did not confirm the presence of nitrogen (N) in the composite, except for the presence of tin (Sn), carbon (C), and oxygen (O).

6. Efficiency and long-term cycling performance for Sn-C anode materials

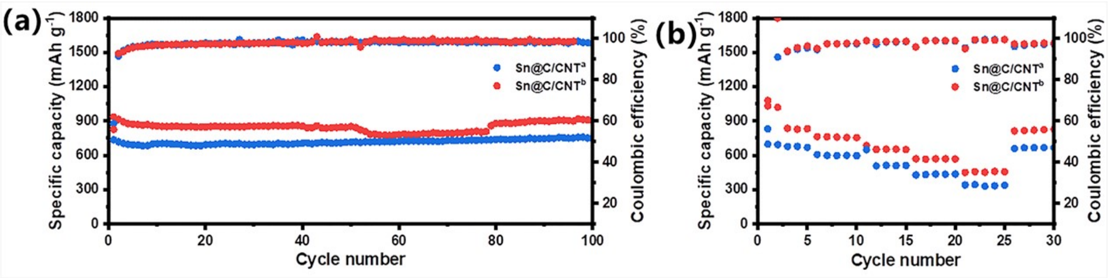

Fig. S9 Efficiency and long-term cycling performance for Sn@C/CNT[a] and Sn@C/CNT[b] anode materials annealed at 700 °C and 900 °C, respectively.

For comparison, two other anode materials, Sn@C/CNT[a] and Sn@C/CNT[b], first annealed at 700 °C and 900 °C, respectively, show inferior long-term cycling performance and rate capabilities, as illustrated in Figure S9. Wherein, The Sn@C/CNT[a] composite exhibits a lower specific capacity of approximately 650 mA h g^{-1}, with a capacity retention ratio of 68.4% and a Coulombic efficiency of nearly 100% within 100 charge/recharge cycles at a current density of 100 mA g^{-1}. In contrast, the Sn@C/CNT[b] composite has a slightly higher specific capacity of around 920 mA h g^{-1}, with a capacity retention ratio of 96.8% and also a Coulombic efficiency close to 100% within the same 100 cycles. However, the apparent decrease in specific capacities from the 52nd to the 79th cycle indicates the unstable redox activity of Sn-Li alloys. This further demonstrates that the annealing temperature significantly impacts their electrochemical performance.

7. Capacity-controlled kinetics analyses for Sn-C anode materials

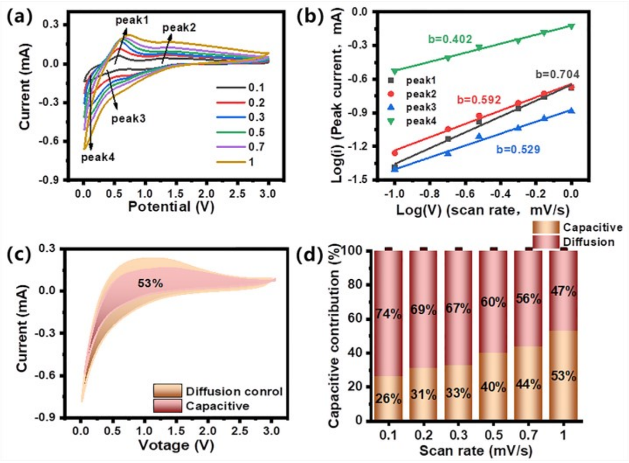

Fig. S10 Capacity-controlled kinetics analyses: (a) LCV curves for Sn@C anode material from a scan rate: 0.1 to 1 mV s^{-1}; (b) Relationship between log (i) versus log (v) for Sn@C/CNT anode material; (c) Capacity-controlled contributions for Sn@C anode material at scan rate of 1 mV s^{-1}; (f) Capacity-controlled contributions for Sn@C anode materials from 0.1 to 1 mV s^{-1}.

Fig. S10 (a) reveals that Sn@C has different redox activity sites than Sn@C/CNT anode materials in LCV testing. The lower adjustable parameters (b=0.705, 0.592, 0.529, and 0.402) corresponding to different current peaks for Sn@C also mean lower capacity-controlled contributions than those of Sn@C/CNT anode materials.

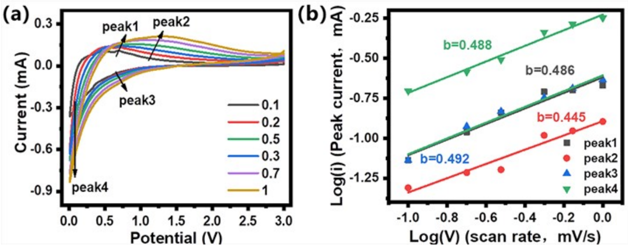

Fig. S11 Capacity-controlled kinetics analyses: (a) LCV curves for Sn*@C/CNT anode material from a scan rate: 0.1 to 1 mV s^{-1}; (b) Relationship between log (i) versus log (v) for Sn@C/CNT anode material

Figure S11(a) shows that the Sn*@C/CNT anode material has more weak redox activity sites compared to the Sn@C/CNT anode materials during LCV testing. The lower adjustable parameters, b, which are 0.486, 0.445, 0.492, and 0.488 corresponding to different current peaks, demonstrate that the Sn*@C/CNT anode materials, originating from commercial Sn nanoparticles, do not exhibit capacity-controlled behavior.
